# First evidence for human occupation of a lava tube in Arabia: The archaeology of Umm Jirsan Cave and its surroundings, northern Saudi Arabia

**DOI:** 10.1371/journal.pone.0299292

**Published:** 2024-04-17

**Authors:** Mathew Stewart, Eric Andrieux, James Blinkhorn, Maria Guagnin, Ricardo Fernandes, Nils Vanwezer, Amy Hatton, Mesfer Alqahtani, Iyad Zalmout, Richard Clark-Wilson, Yahya S. A. Al-Mufarreh, Mahmoud Al-Shanti, Badr Zahrani, Abdulaziz Al Omari, Faisal Al-Jibreen, Abdullah M. Alsharekh, Eleanor M. L. Scerri, Nicole Boivin, Michael D. Petraglia, Huw S. Groucutt

**Affiliations:** 1 Australian Research Centre for Human Evolution, Griffith University, Brisbane, Australia; 2 Extreme Events Research Group, the Max Planck Institutes of Geoanthropology, Chemical Ecology, and Biogeochemistry, Jena, Germany; 3 Department of Archaeology, Durham University, Durham, United Kingdom; 4 Human Palaeosystems Group, Max Planck Institute of Geoanthropology, Jena, Germany; 5 Department of Archaeology, Max Planck Institute of Geoanthropology, Jena, Germany; 6 Department of Bioarchaeology, Faculty of Archaeology, University of Warsaw, Warszaw, Poland; 7 Arne Faculty of Arts, Masaryk University, Brno, Czechia; 8 Climate Change and History Research Initiative, Princeton University, Princeton, New Jersey, United States of America; 9 Institute for Archaeological Sciences, Department of Geosciences, University of Tübingen, Tübingen, Germany; 10 Department of Anthropology, University of Pittsburgh, Pittsburgh, Pennsylvania, United States of America; 11 Palaeontology Division, Directorate of Geological Survey, Survey and Exploration Centre, Saudi Geological Survey, Jeddah, Saudi Arabia; 12 Museum of Palaeontology, Research Museum Centre, Ann Arbor, Michigan, United States of America; 13 Centre for Quaternary Research, Department of Geography, Royal Holloway, University of London, Surrey, United Kingdom; 14 Geotourism Department, Saudi Geological Survey, Jeddah, Saudi Arabia; 15 Heritage Commission, Ministry of Culture, Riyadh, Saudi Arabia; 16 Department of Archaeology, College of Tourism and Archaeology, King Saud University, Riyadh, Saudi Arabia; 17 Department of Classics and Archaeology, University of Malta, Msida, Malta; 18 Institute of Prehistoric Archaeology, University of Cologne, Cologne, Germany; 19 School of Social Science, University of Queensland, St Lucia, Australia; 20 Griffith Sciences, Griffith University, Brisbane, Australia; 21 Human Origins Program, Smithsonian Institution, Washington, D.C., United States of America; Tel Aviv university, ISRAEL

## Abstract

Recent advances in interdisciplinary archaeological research in Arabia have focused on the evolution and historical development of regional human populations as well as the diverse patterns of cultural change, migration, and adaptations to environmental fluctuations. Obtaining a comprehensive understanding of cultural developments such as the emergence and lifeways of Neolithic groups has been hindered by the limited preservation of stratified archaeological assemblages and organic remains, a common challenge in arid environments. Underground settings like caves and lava tubes, which are prevalent in Arabia but which have seen limited scientific exploration, offer promising opportunities for addressing these issues. Here, we report on an archaeological excavation and a related survey at and around Umm Jirsan lava tube in the Harrat Khaybar, north-western Saudi Arabia. Our results reveal repeated phases of human occupation of the site ranging from at least the Neolithic through to the Chalcolithic/Bronze Age. Pastoralist use of the lava tube and surrounding landscape is attested in rock art and faunal records, suggesting that Umm Jirsan was situated along a pastoral route linking key oases. Isotopic data indicates that herbivores primarily grazed on wild grasses and shrubs rather than being provided with fodder, while humans had a diet consistently high in protein but with increasing consumption of C_3_ plants through-time, perhaps related to the emergence of oasis agriculture. While underground and naturally sheltered localities are globally prominent in archaeology and Quaternary science, our work represents the first such combined records for Saudi Arabia and highlight the potential for interdisciplinary studies in caves and lava tubes.

## Introduction

Intensified field research in northern Arabia over the last decade has highlighted the richness and diversity of the region’s archaeological and palaeontological records. Human occupation in northern Arabia during the Pleistocene was sporadic and seemingly linked to periods of improved climate, though by the Holocene people were able to more consistently settle the region through dry intervals [1, 2]. The proliferation of archaeological sites in the Holocene has been interpreted as reflecting population growth in the region, spurred by the onset of the Holocene Humid Period (HHP) at around 10,600 years before present (BP). This was followed by the introduction of domestic livestock, and later by the development of water-harnessing technologies (e.g., wells, dams) and oasis agriculture in the Bronze Age, when arid conditions returned [2]. Many of the features that define the Neolithic elsewhere, such as sedentism, pottery, and agriculture, are notably absent from northern Arabia until the Bronze Age. As such, we follow previous works [3] in classifying the ‘pre-Neolithic’ as the period preceding the introduction of livestock (before ca. 8,000 years BP) but for which there are apparent cultural links to Neolithic groups in the Levant, and the subsequent ‘Neolithic’ as the period following the introduction of livestock (after ca. 8,000 years BP) and characterized by highly mobile herders that retain hunting in their cultural subsistence practices.

Evidence for pre-Neolithic occupation is recorded in the rock art of northern Arabia. This includes hunting scenes superimposed by depictions of livestock herds, as well as reference to the HHP in the depiction of fauna (e.g., lesser kudu, African wild ass) that today do not inhabit true deserts [4–6]. Pre-Neolithic artefacts have also been recovered, though such findings are restricted to just a handful of sites. In the Jubbah Basin, lithics with similarities to the Levantine Geometic Kebaran were found deposited on sediments dated to ca. 12,250 years BP at Al-Rabyah [7]. However, detailed geochronological analysis suggests that this may reflect a minimum age [8], with similar assemblages in the Levant dating to ca. 18,000–16,250 years BP [9]. Assemblages with similarities to the Pre-Pottery Neolithic (PPN; dated to ca. 12,175–8,450 years BP in the Levant) have been documented at Jebel Qattar 101 [10] and Jebel Oraf [11] with finds at the former putatively associated with an adjacent palaeolake dated to 8,978–7,900 years BP. Just south of the Nefud Desert at the recently discovered site of Sahout, a backed bladelet of a type common in the Levantine Natufian (where this period dates to ca. 14,900–11,750 years BP), albeit also known from the PPNA (ca. 12,175–11,000 years BP), was found [12]. The presence of archaeological deposits at the site dating to the Neolithic, but also to earlier periods (ca. 13,400–8,800 years BP), and their association with large naturalistic camel engravings, supports earlier hypotheses that this rock art tradition may pre-date the Neolithic [12].

Neolithic occupations in northern Arabia are better represented. This includes a rich rock art record in which the herding of cattle and caprids is commonly depicted [4, 13]. Hearth sites are also a common feature of the early Neolithic landscape of northern Arabia, often occurring in high numbers alongside ancient lake deposits. For example, at Jebel Oraf, 170 hearths have been documented, the majority of which date to roughly between 7,300 and 7,000 cal. years BP, but also extending up until the recent period, indicating a recurrent and long-term use of the basin [3]. At Alshabah in the western Nefud Desert, 125 hearths were documented, with the dating of three of these producing ages between ca. 7,300 and 6,500 cal. years BP [14]. The abundant rock art, hearth deposits, and associated wild (e.g., gazelle, ostrich) and domesticated faunal remains (e.g., cattle, caprids) suggest that regions like the Jubbah and Alshabah basins were important foci in the landscape for early pastoralists and their herds.

More recently, efforts to document, excavate, and date the plethora of megalithic stone structures that can be found scattered across the deserts have revealed that these too formed part of the pre-Neolithic, Neolithic, and later period landscapes of northern Arabia [15–22]. Of these, the famous hunting mega-traps—commonly known as ‘desert kites’—may be the oldest, as suggested by recent work in southern Jordan indicating their construction by as early as ca. 10,000 years BP [23–25]. Although very few of these structures have been directly dated, it appears that they may have been built and in use for millennia [26, 27], including into historic times, as implied in early ethnographic accounts that recall gazelle hunts in Jordan and Syria seemingly employing such structures [26, 28, 29].

The next oldest structures appear to be the circular dwellings with upright stones and the large rectangular structures called ‘mustatils’, both dating from around 7,200 years BP [16, 17, 20]. The latter appears to have had a ritualistic purpose, as suggested by the intentional placement of selected wild and domestic animal remains—namely bucrania—as well as orthostats and small fires within the structure’s chambers [17, 30]. More than their ritual purpose, it has been suggested that mustatils were important for maintaining socio-economic and cultural links between families and the wider community through activities such as feasting, as well as having functioned as territorial markers [21].

Another impressive feature of the region are the ‘funerary avenues’ which comprise long-distance pathways flanked by pendant-shaped structures that radiate out from major oases, and which may date from as early as ca. 5,600 years BP [31]. The fact that these ‘avenues’ link together major water sources, while the earlier mustatils are often oriented towards water, suggests that these stone structures may have played an important role in pastoralist social, economic, and cultural lifeways over millennia. In addition to these, a variety of other structures such as trapezoidal platforms [19] and thousands of burial cairns have also been documented [16, 32].

Taken together, these findings have highlighted the dynamism of the Holocene—and possibly terminal Pleistocene—archaeological record of northern Arabia. Despite these efforts, however, the exact timing and nature of the various occupations in northern Arabia, and their connections with groups in the nearby Levant, remain poorly understood. A principal reason for this relates to the poor preservation of organic remains (e.g., bone, pollen, phytoliths) in arid environments [33, 34]. This is well illustrated at the Oraf 2 hearth site, where of the >1800 bone fragments less than 1% were identifiable to a taxon [3], and where almost no macrobotanical remains were recovered despite strong use-wear evidence for the on-site processing of plant remains [33]. Wind erosion, heat exposure, and high amplitude temperature fluctuations all serve to degrade and fragment bones and other organic remains in Arabia [34]. Such processes are even problematic for remains interred within structures. However, some recent excavations have uncovered exceptionally well-preserved faunal remains due to their positioning under rocky outcrops that serve to protect the remains from the elements [17, 30].

**Fig 1:**
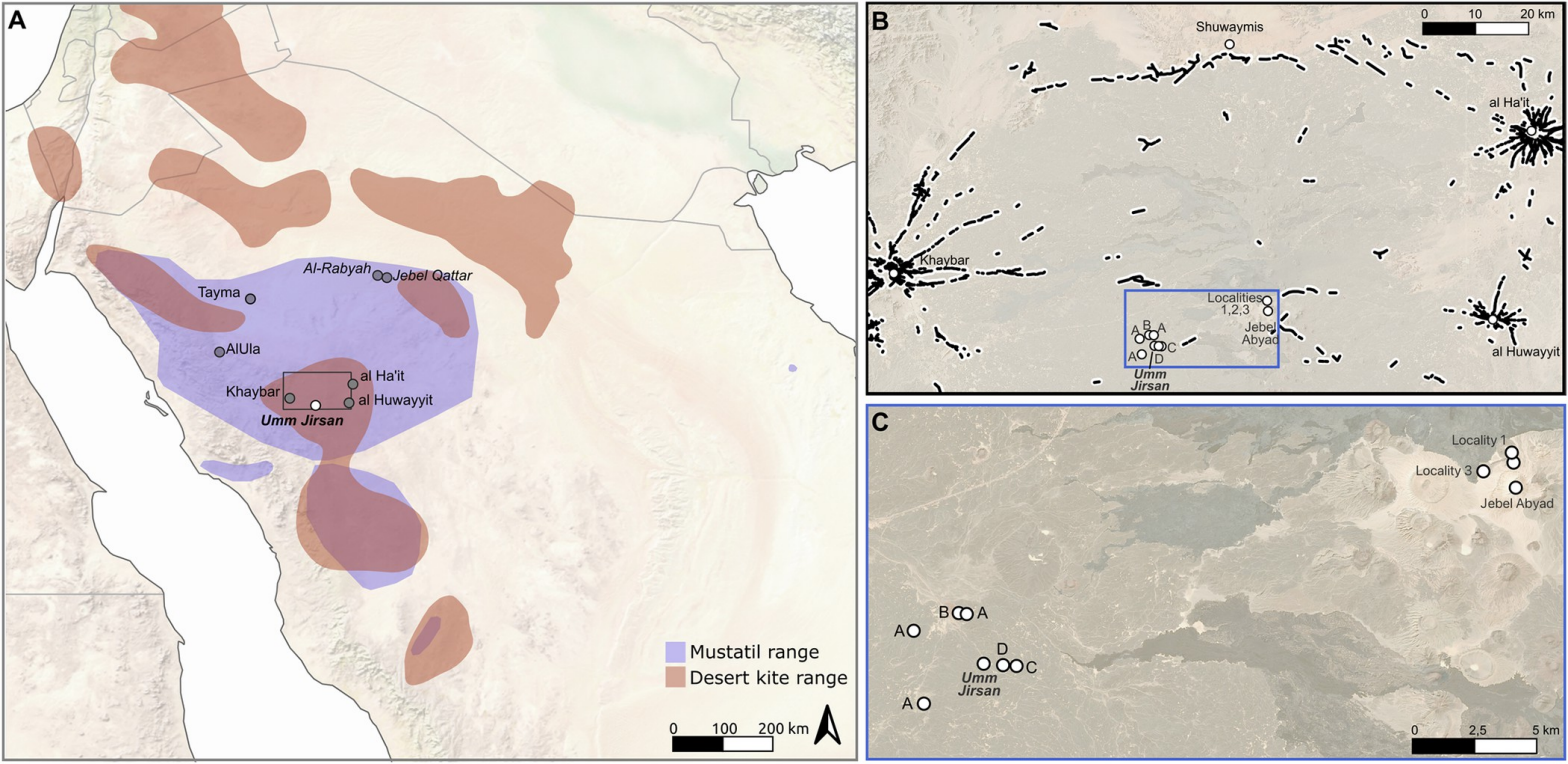
Map of northern Arabia and key sites. (A) Map showing the location of Umm Jirsan and key sites mentioned in the text, as well as the range of mustatils and desert kites [16, 17, 20, 24, 27, 39] and (B) pendant tombs (black lines and dots) in the Harrat Khaybar [31]. (C) Location of newly identified archaeological sites are shown in the inset: (A) stone structures (25.6087 N, 39.7502 E, 25.6020 N, 39.7289 E, and 25.5728 N, 39.7330 E); (B) ‘bow-tie’ shaped structure (25.6091 N, 39.7470 E); (C) lava tube collapse with rock art (25.5879 N, 39.7702 E); and (D) Umm Jirsan D area. Made with Copernicus COP-DEM 30 m and “Sentinel-2 cloudless—https://s2maps.edu by EOX IT Services GmBH (contains modified Copernicus Sentinel data 2020)”, under CC BY 4.0.

**Fig 2:**
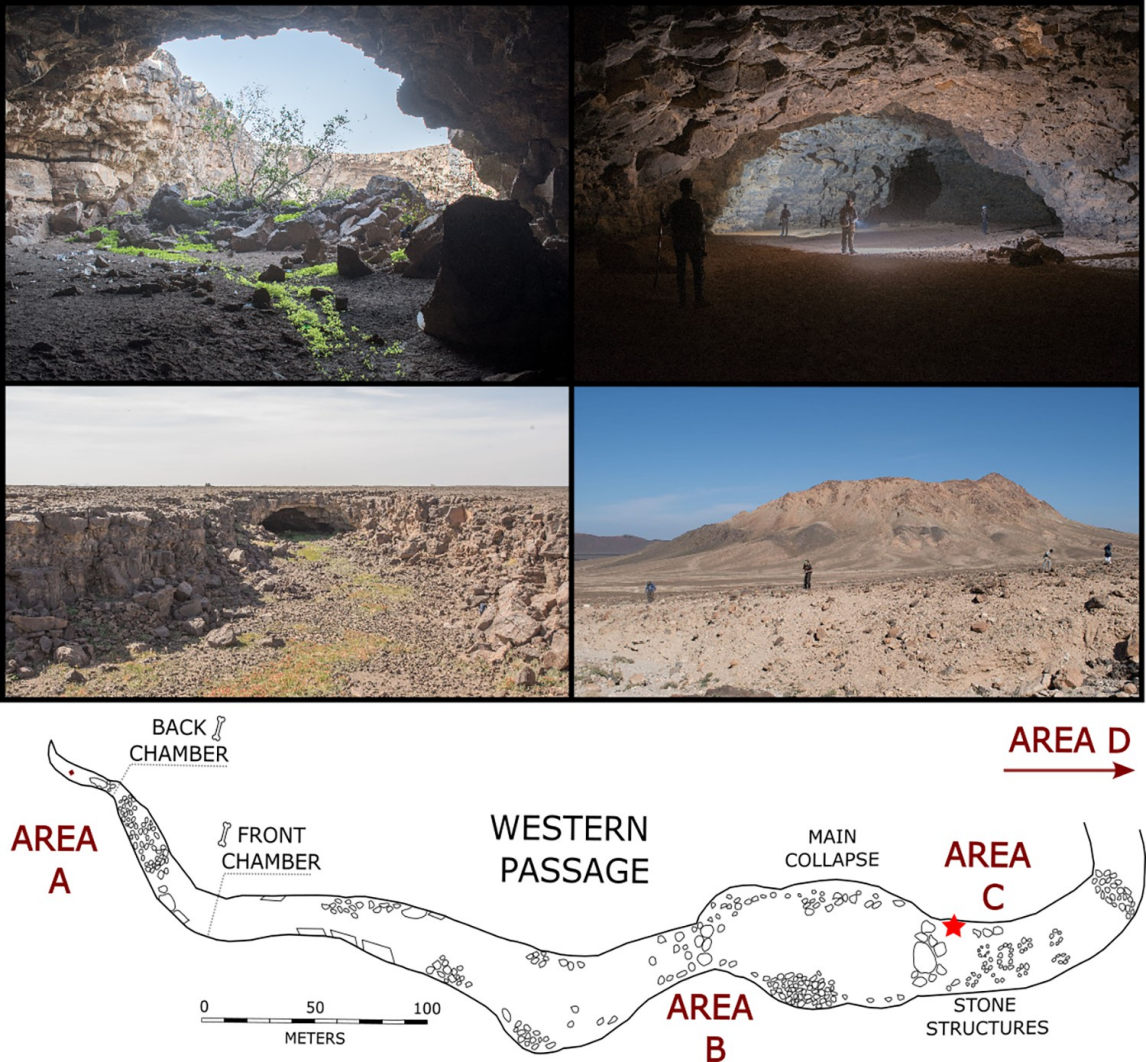
Photographs of the Umm Jirsan Cave and interior sections of the lava tube. Top left, looking out of entrance to Umm Jirsan (Trench 1 just out of view on right). Top right, inside lava tube beyond Trench 1. Middle left, another example of a lava tube near Umm Jirsan. Middle right, Jebel Abyad area with obsidian clasts and lithics, beneath obsidian outcrops. Bottom, simple plan of the Umm Jirsan lava tube system with red star indicating the location of the Trench 1 excavation [modified from 40].

To that end, our fieldwork was redirected to investigate caves and other underground settings where organic remains have a better chance of survival. Despite long-standing explorations of caves and lava tubes in northern Arabia [35–38], often for their potential as tourism show caves, none have been subjected to systematic archaeological survey or investigation. Here, we report our work on the Umm Jirsan lava tube (25.5888 N, 39.7570 E; WGS84), approximately 125 km north of Medina. Umm Jirsan Cave represents both one of the first documented underground archaeological sites in the interior of Arabia and one of the few sites in Saudi Arabia that has been dated to the early to mid-Holocene (Figs 1 and 2).

### Harrat Khaybar and Umm Jirsan Cave

Umm Jirsan is located in the Harrat Khaybar, a volcanic area comprised of *harrats* (singular: *harra* [حَرَّة], Arabic plural: *harrat)* in north-western Saudi Arabia. Early work by Gilmore and colleagues [41, p. 13] reported archaeology ranging from the Lower Palaeolithic through to the Neolithic, the latter including “tabular flint scrapers, blades, bifacial retouch, ground stone, trianguloids… and “T” shaped notched tools.” More recently, aerial and remote sensing work has documented hundreds of Neolithic and Bronze Age megalithic structures [22, 42, 43]. This work has demonstrated the rich archaeological record of the area. However, with only limited excavations and absolute dating, it has remained challenging to build a detailed view on human prehistory in the area.

Annually the area receives little rainfall (<100 mm), soil cover is sparse, and vegetation consists mostly of xeromorphic dwarf shrublands [44]. Despite the limited rainfall, the *harrats* often have well-developed wadi systems that feed into major oases, promoting aquifer recharge and the activation of springs [18]. During the more humid periods of the Pleistocene and Holocene, depressions bordering the lava fields would have boasted freshwater ponds and lakes, generating wetlands along these drainage courses that would have promoted vegetation growth, increased biodiversity, and facilitated human and animal movements [45].

Umm Jirsan is currently the longest reported lava tube in Arabia in terms of the horizontal length of passages, at 1481 metres (m), and has a typical passage height of 8–12 m and maximum passage width of 45 m [36]. The lava tube consists of three segments separated by two large collapses. Previously, entry into the cave was rather difficult, but in 2017 a wall was built by the Saudi Commission for Tourism and National Heritage around the main collapse entrance and a large staircase inserted. From this collapse, the huge western entrance leads to a large passage, at the end of which the passage rises and then soon becomes blocked with boulders.

Here, and elsewhere throughout Umm Jirsan, massive caches of bone can be found, and we previously reported on the excavation of one such cache located in the back chamber of Area A (Fig 2) [40]. Taphonomic and ethological analysis revealed that the Area A bone assemblage is the product of striped hyena (*Hyaena hyaena*) bone accumulating and denning behaviours. The material is exceptionally well-preserved and preliminary radiocarbon dating revealed that the fossils date as far back as 7,000 cal. years BP. From the excavation, remains of microfauna (e.g., lizards, birds, hare, rock hyrax), carnivores (e.g., wolf, hyena), various ungulates (e.g., gazelle, caprid, cattle, camel, and equids), and two human cranial fragments were recovered, the latter likely resulting from striped hyena’s ability to loot human grave sites. In addition to these two specimens, seven human cranial fragments were recovered from elsewhere in Umm Jirsan: four from the Area A front chamber; one from nearby Trench 1 in Area C; and another further along the eastern passage between Area C and Area D (Fig 2) [see 40].

The eastern passage from the main collapse entrance is also very large, around ten metres high and 30 metres wide. Various circular stone structures—as well as apparent rectangular structures and a stone wall found elsewhere at Umm Jirsan—attest to human use of the lava tube at some point in the past (S9 Fig in S1 File) [36]. Here, we report on a second excavation undertaken in the eastern passage of the lava tube, supplemented with the discovery of lithic artefacts and rock art in the surrounding region, as well as isotopic data obtained from human and faunal remains recovered from throughout Umm Jirsan.

## Methods

### Survey and excavation

We surveyed Umm Jirsan Cave with an interdisciplinary team to evaluate visible archaeological, palaeontological, and sedimentary materials and identify areas possibly preserving sub-surface material. For ease of description, several areas of the cave have been labelled with simple codes following our earlier exploration of the cave [40] and shown in Fig 2.

A pit previously dug along the northern wall of the entrance to Area C, possibly for the purposes of looting or reaching water, revealed that lithic artefacts were present in the sediments. Therefore, we decided to place a 1 x 1 m test excavation in an undisturbed area adjacent to the pit (Trench 1). We used a single context excavation method, differentiating discrete excavation units (XUs) based on the sedimentology, but subdividing them into 10 cm thick arbitrary XUs where necessary to help control artefact provenance within a given deposit. A total station was employed with EDM software [46] to record the position of large artefacts (>20 mm), as well as spatial extents of each deposit, the location of sediment samples recovered, and key characteristics of the trench location within the cave. All excavated sediments were sieved through 5 mm mesh to enhance artefact recovery, with further sieving of spoil from the pit to collect further finds. Samples for luminescence dating were recovered from the sediment section in opaque metal tubes with associated bulk samples.

In addition to the excavation, the area to the east of Umm Jirsan was surveyed. Where archaeological materials were observed, their location was recorded using GPS devices, photographs taken, and representative samples collected. In the case of rock art, multiple photos were taken and then the ImageJ © plugin DStretch was used to aid visibility. DStretch uses a number of pre-set algorithms that allow reproducible modification of contrast, hue, and colour space [47]. Although the DStretch plugin is designed to enhance the visibility of rock paintings, several settings also produce good results for rock engravings. In addition, photographs were modified using Adobe Lightroom to enhance visibility of the faded engravings.

### Lithic and faunal analysis

Lithics were analysed to record their raw material characteristics and basic typo-technological features. Photographs and illustrations were taken of selected pieces. The methodology follows that previously utilised by our project in the region [48, 49]. Samples of obsidian were collected at identified raw material outcrops in the survey east of Umm Jirsan and used for knapping experiments to understand the properties of the rock [see also 50, 51]. While this rock has previously been described as obsidian [49, 50], it has not yet been geochemically analysed in detail, and appears rather opaque and may well be characterised as rhyolitic obsidian or rhyolite/obsidian. Hereafter we refer to the material as obsidian but note the need for future studies to elucidate its characteristics.

Faunal bones recovered from Trench 1 and the spoil heap (NRSP = 653) were identified to the lowest taxonomic level, facilitated by key literature [e.g., 52, 53] and osteological collections housed at the Royal Museum for Central Africa, Belgium. Maximum specimen length and width were collected using digital callipers and additional morphometric measures taken following Harrison and Bates [52] and von den Driesch [54] to assist in taxonomic attribution. Well-established quantitative units were used to present the results: number of recovered specimens (NRSP); number of identified specimens (NISP); minimum number of elements (MNE); and minimum number of individuals (MNI). MNE and MNI were calculated using a zonation system adapted from Dobney and Rielly [55]. Unlike MNE, MNI was calculated by taking into consideration the laterality (left, right) of the element and age of the individual. Normalized values (%NISP, %MNE) were also calculated following Binford [56].

Given the limited assemblage, all specimens were placed into one of two size classes of animal; microfauna (<1 kg; e.g., rodents, birds) and macromammals (>1 kg; gazelle, caprids, equids). All specimens were assessed for bone surface modifications (e.g., weathering, gnaw marks) by eye and light microscopy following established methods [e.g., 57]. Long bone fracturing (fracture angle, edge, and outline) and circumference completeness were recorded following Villa and Mahieu [58] and Bunn [59], respectively. Mandible and maxilla fragmentation and degree of digestion of rodent in-situ molars and isolated incisors were recorded following the criteria described by Fernández-Jalvo et al. [60]. For full discussion of the methods used, see Stewart et al. [61].

### Optically stimulated luminescence (OSL) dating

**Fig 3:**
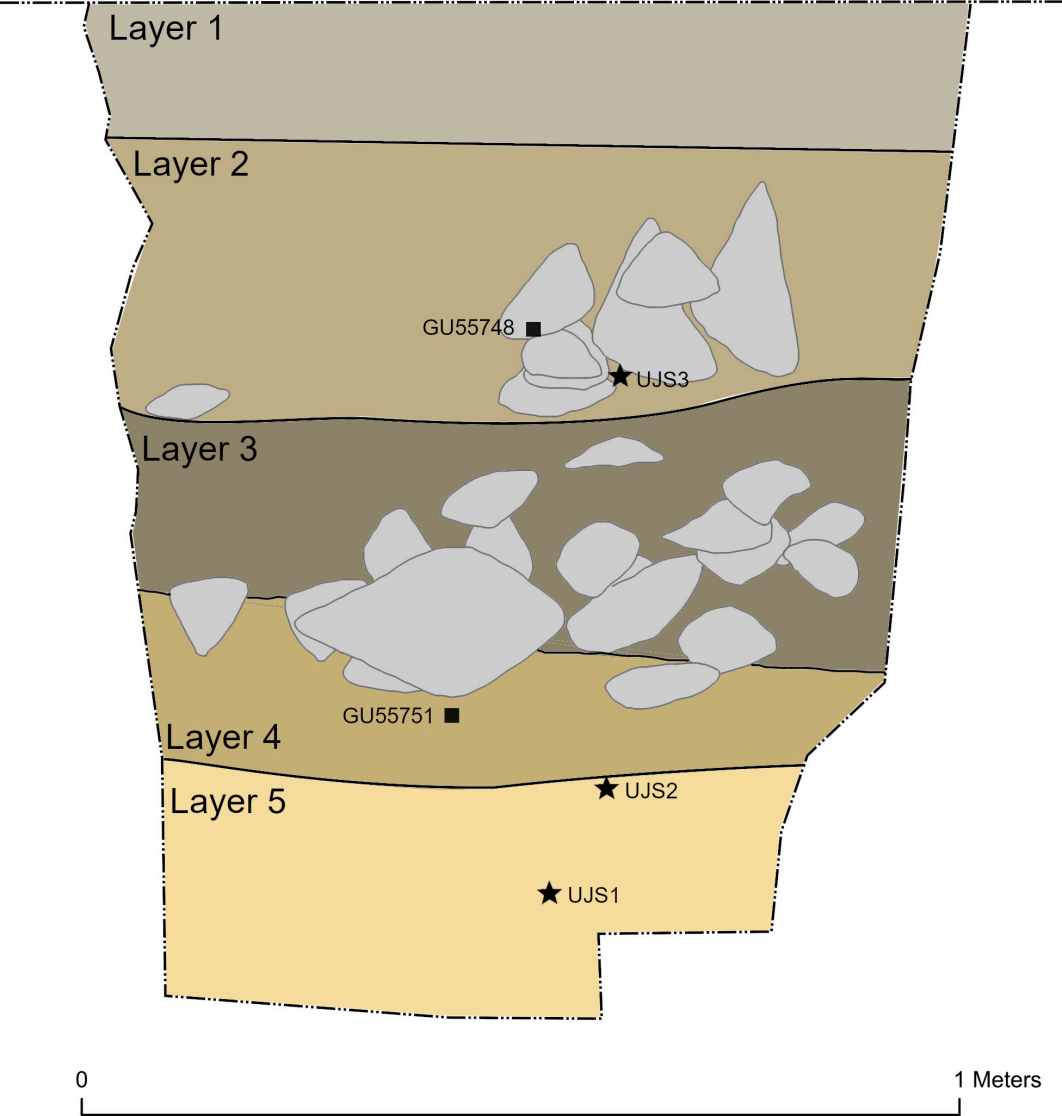
Trench 1 stratigraphy. Schematic illustration of stratigraphic sequence of Trench 1 at Umm Jirsan showing the five major layers and the location of the three luminescence samples (black stars).

Three samples (UJS1, UJS2, and UJ3) were collected from the excavated trench at depths of 1.04 m, 0.94 m, and 0.45 m from the surface, respectively (Fig 3). The samples were sent to the Royal Holloway Luminescence Laboratory, Royal Holloway University of London, to be prepared under subdued red-light conditions. The outer, light-exposed portions of each sample were removed and used for environmental dose rate measurements and estimation of the sample’s moisture content. The remaining sediment was treated with hydrochloric acid (1M HCl) and hydrogen peroxide (H_2_O_2_) to remove carbonate and organic matter respectively. The samples were wet sieved to the 150–180 μm fraction size. Quartz and K-feldspars were extracted using density separation at 2.58 g/cm^3^, 2.62 g/cm^3^, and 2.70 g/cm^3^. Full details of the equipment used and results can be found in the S1 File.

### Radiocarbon dating

Radiocarbon dating of bone samples was carried out at the Scottish Universities Environmental Research Centre (SUERC) Radiocarbon Dating Laboratory. Collagen was extracted using a modified Longin method and the dried extract was subject to combustion, reduction, and AMS measurement following established methods [62, 63]. Charcoals were subject to an acid-base-acid cleaning protocol prior to radiocarbon measurement following Dunbar et al. [62].

### Isotopic analysis and Bayesian modelling

Collagen extracted for radiocarbon dating was also subject to stable isotope analysis at SUERC following Dunbar et al. [62]. Ancillary carbon (δ13C) and nitrogen (δ15N) stable isotope ratios are expressed using delta notation relative to the Vienna Pee Dee Belemnite (VPDB), Atmospheric Nitrogen (AIR) standards, respectively. Laboratory uncertainty values were calculated as ±0.1‰ for δ13C and ±0.2‰ for δ15N.”

A small number of faunal remains recovered from throughout Umm Jirsan, though primarily from the Area A excavation and reported in Stewart et al. [40, Table 1], were assessed for their δ^13^C and δ^15^N isotope values (n = 9). This comprises teeth (dentine) from two gazelle (*Gazella* sp.), a single caprid (*Capra* sp.), a single aurochs/cattle (*Bos* sp.), and five wild ass/donkey (*Equus* sp.). Of the nine human cranial fragments recovered from Umm Jirsan, four were radiocarbon dated and have corresponding stable δ^13^C and δ^15^N isotopic data. One of these originated from the original Area A excavation (UJS-2019-1031), two were discovered in the Area A front chamber (UJS-2019-200040, UJS-2019-200042), and the fourth from Area C near the new Trench 1 (UJS-2019-100013) (see Fig 2).

The faunal remains range in age from ca. 400 to 4,100 cal. years BP, whereas the human remains range from ca. 150 to 6,000 cal. years BP [40]. While these finds were not recovered from the Trench 1 excavation directly, given their proximity to the trench—all from within a few hundred meters of Trench 1—and the broad and overlapping ages, they provide a through time record of climatic and dietary change over a period directly relevant to the findings from Trench 1.

Local annual rainfall and temperature is correlated with the prevalence of plant photosynthetic pathways in the grass cover of arid regions [64–66], with typically a greater presence of C_4_ plants in hotter and drier settings. Using δ^13^C values from the skeletal remains of grazing herbivores, which randomly sample from available grasses and are not subject to foddering by humans, it is possible to reconstruct past grass covers [67]. In addition, aridity is also correlated with δ^15^N values in plants, with higher aridity levels resulting in higher δ^15^N values for plants and consequently for grazers [68, 69]. Therefore, there is an expected relationship between δ^13^C and δ^15^N values for grazers feeding on wild grasses.

Bayesian dietary estimates were made using the R-based software ReSources, an upgraded version of the Bayesian mixing model FRUITS [70, 71]. To model herbivore C_3_ vs. C_4_ intakes, a two-member model was employed with reference δ^13^C values of -25.2‰ and -11‰, respectively. These correspond to averages of modern grasses published by Cerling and colleagues [67] shifted by 1.5‰ to account for fossil fuel contributions to atmospheric CO_2_ δ^13^C values. An isotopic offset of 5±0.5‰ between plant diets and herbivore bone collagen was included in the model. A similar model was implanted for human estimates of C_3_ vs. C_4_ protein intakes. This assumes that there is a negligible isotopic difference in human δ^13^C values when consuming directly C_3_ or C_4_ plants or animals fed on these. Finally, for human estimates of plant vs. animal protein intakes, a two end-member model was implemented with an δ^15^N offset of 5.5±0.5‰ between diet and consumer [72]. Reference plant and animal δ^15^N values were 6.8‰ and 10.3‰, respectively. The latter correspond to selected herbivore average and to obtain a plant reference an offset of 3.5‰ was subtracted.

To produce a smooth curve for human δ^13^C values though time, the Bayesian software PlotR was employed. This uses a thin plate spline approach to produce smoothed curves following Wood [73]. The curve was generated using 4 basis functions with a uniform distribution for x-values (95% calibrated ranges for individual radiocarbon dates). Full model description can be found on the Pandora & IsoMemo software platform [74].

### Ethics statement

All necessary permits were obtained for the described study, which complied with all relevant regulations.

The nine human skeletal remains from Umm Jirsan mentioned in the described study are presently housed at the Max Planck Institute of Geoanthropology, Germany. Once all analyses are finalized, these specimens will be returned to the Saudi Heritage Commission, Saudi Arabia, for permanent storage. Collection and analysis of these specimens were done under a permit granted by the Saudi Heritage Commission.

Specimen numbers: UJS-2019-1031, UJS-2019-100013, UJS-2019-200040, UJS-2019-200041, UJS-2019-200042, UJS-2019-200043, UJS-2019-200044, UJS-2019-200045, UJS-2019-300027.

## Results

### Umm Jirsan archaeology

Archaeological remains were found in various parts of Umm Jirsan (Fig 1). We describe these from west to east. At the far western end of the lava tube, Area A consists of the large bone accumulation that was published in Stewart et al. [40], where, among the bones, fragments of cloth and worked wood were found. Moving east, three lithics were found just inside the western entrance to the lava tube (Area B). One large lithic (360 g) and a fresh obsidian flake were found on the entrance slope down into the tube, and two smaller flakes were slightly further underground at the base of the passage where a small streambed has incised the floor. At least one semi-circular structure is located against the northern wall of the western entrance, the age of which remains unknown. Occasional lithics were also found at the base of the main entrance collapse (i.e., at the base of the newly built stairs).

**Fig 4:**
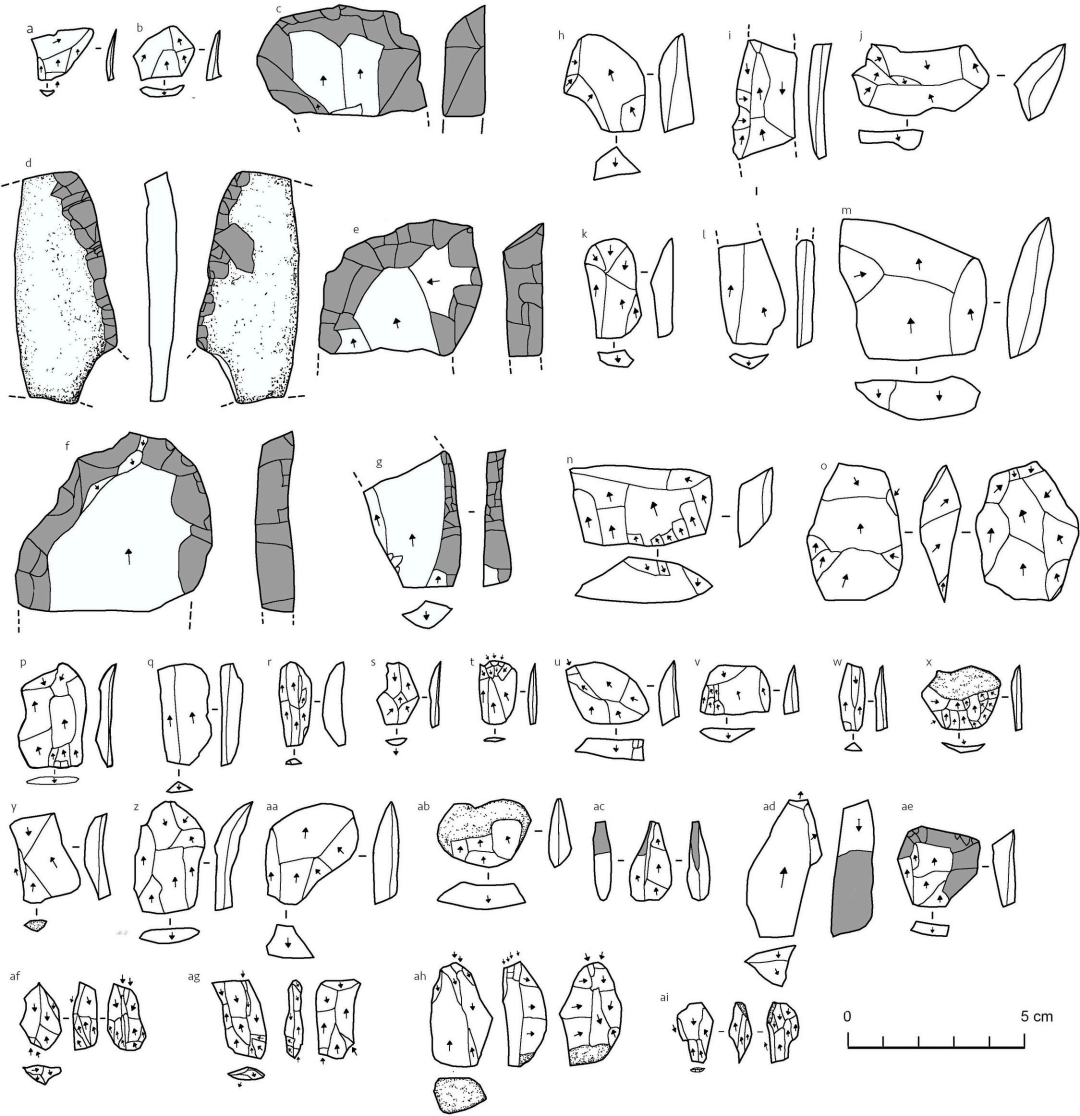
Umm Jirsan lithic artefacts. A-G: Area C looters pit spoil heap, H-O: Trench 1, P-AI: Area D. Grey areas are retouched. a,b,h,k,n,n,p-ab = flakes, c,d-f, ac-ae = retouched flakes (including burins: ac and ad), cores: o, af-ai.

In the eastern entrance (Area C) we retrieved several lithics located in the spoil heap of the previously dug hole. Sieving the spoil heap produced 20 lithics with an average weight of 11.4 g from approximately 375 litres of sediment (Fig 4A–4G). Eighteen of these are of different forms of dark green obsidian, with the other two being of chert and basalt. Most of the lithics consist of small flakes, but interesting pieces include three similar retouched flakes (Fig 4C, 4E and 4F), which are invasively retouched distal fragments of large flakes. The repeated shape suggests that flake breakage to produce short and wide flakes was deliberate and a way to produce thick squat ‘scrapers’. The chert piece is a very thin plaquette (Fig 4D), laterally broken and finely retouched along one margin. The chert must have been imported from quite a considerable distance, probably from the north [75]. Semi-circular stone structures and pits are also present in the Area C entrance (S9 Fig in S1 File). Due to the volume of stone, dust, and bird droppings, it is hard to distinguish these features clearly, but a large proportion of the area is clearly anthropogenically modified.

Following the eastern passage underground eventually leads to another collapse entrance (Area D; 25.58823 N, 39.7648 E). Just inside the entrance, several circular structures (essentially cleared areas) are present (S9 Fig in S1 File). In the eastern part of this collapse, a lithic assemblage was identified from an area measuring approximately 6 x 6 m (Fig 4P–4Ai). This Area D lithic scatter included six cores (Fig 4Af–4Ai), 91 flakes, eight retouched flakes, and 28 chips and chunks (n = 133). Most of the assemblage (86%) consists of obsidian of varying colours from green, to grey, and black, with greenish colour being the most common. Aside from a single rhyolite artefact, the rest of the assemblage consists of quartz. The lithics are in a fresh condition and are mostly very small, with an average weight of 1.8 g, with the smallest weighing only 0.1 g. Indeed, aside from a single retouched flake at 18.1 g and a quartz core at 17.4 g, all lithics from the site weigh less than 10 g and 75% are less than 2 g. The assemblage reflects the use of bipolar reduction to produce small and elongated flakes. There is also some use of multiplatform methods, likely with free-hand percussion. Three burins were identified (Fig 4Ac and 4Ad), and retouched forms display relatively simple and informal retouch, focussed on the lateral margins. Overall, the weathering and technology of these lithics suggest that they are younger than those from Area C. While not confirmed by absolute dating, it is possible that the Area D lithics date to a period such as the Chalcolithic or Bronze Age. In addition to the lithics, a piece of pottery and three ostrich eggshell fragments were recovered among the Area D lithic scatter.

### Trench 1 excavation and dating

A 1 x 1 m trench (Trench 1) was excavated a few metres east of the previously dug pit (Figs 2 and 3) resolving five major layers excavated across thirteen excavation units (XUs), reaching a total depth of 114 cm.

Surface deposits (Layer 1; XU 001) were excavated as a single 18 cm thick layer, comprising dry and dusty greyish brown silty sands that were distinguished from underlying deposits due to differences in moisture content. Layer 2 (XU 002–005) comprised a ~30 cm thick deposit of increasingly moist, moderately compacted pale yellowish-brown silty sand. Potentially bioturbated deposits observed at the interface with the underlying sediment unit were constrained in a single XU (XU 006), which comprised a soft dark grey deposit containing charcoal and rootlets. Layer 3 (XU 007–009) comprised a ~25 cm thick deposit of mid greyish brown silty sands supported by pebble to cobble sized clasts. Underlying this, Layer 4 (XU 010–011) was an approximately 20 cm thick deposit of pale to dark yellowish brown silty sand with some clast support present in XU 010. The basal deposits uncovered (Layer 5; XU 012–013) were observed to a minimum thickness of ~25 cm and composed of a firm pale brownish yellow silty clay with sub-angular geometric texture.

Animal bone fragments were common throughout the sequence but were fragile and generally turned to powder as soon as they were touched. Charcoal was also relatively common, and four samples were selected for radiocarbon dating (Table 1). The sediment sequence suggests early sediment formation in very low, humid depositional settings (Layers 5–4), interrupted by potential cave wall collapse or increased clast mobility within the lava tube (Layer 3), with a return to lower energy but drier depositional conditions towards the top of the sequence (Layers 2–1).

**Table 1:** AMS radiocarbon ages from Umm Jirsan Trench 1 calibrated using Calib (v. 8.20) and using IntCal20 [76]. Radiocarbon dating analysis conducted at the SUERC Radiocarbon Laboratory, University of Glasgow.

Lab code	XU and depth	Material	AMS date (bp)	Cal. BP 95.4% (2 σ)
GU55748	004 (0.38 m); Unit 2	Charcoal	5074 ± 23	5746–5832 (63.8%) 5840–5904 (36.2%)
GU55749	008 (0.56 m); Unit 3	Charcoal	Failed	
GU55751	011 (0.79 m); Unit 4	Charcoal	9174 ± 25	10,243–10,411 (96.8%) 10,468–10,481 (3.2%)
GU55750	011 (0.82 m); Unit 4	Charcoal	Failed	

The upper part of the trench dates to the mid/late Holocene with the OSL sample from XU 005 (Layer 2) and radiocarbon sample from XU 004 both giving ages of ca. 5,800 cal. years BP (Tables 1 and 2). The main occupation horizon, XU 011 (Layer 4), has a radiocarbon date of ca. 10,300 cal. years BP. OSL ages from just beneath this were ca. 8,700 years BP for XU 012 and ca. 9,800 years BP for XU 013 (Layer 5). There is a slight inversion with regards to the lower OSL and radiocarbon ages, so the age of the main occupation layer may conservatively be described as more than the ca. 6,000 years BP and less than ca. 10,300–8,700 years BP. Given the depth and presence of another episode of roof fall between XU 011 and the upper dates, we suggest the most likely age range is towards the older end of that range, at approximately 10,300–8,700 years BP. Given the thin concentration of lithics in the main occupation horizon, and the fact that most of the radiometric age estimates are in stratigraphic order, we assume that we are dealing with a basically in-situ deposit. Radiocarbon ages from faunal remains obtained from earlier works are provided in Table 3.

**Table 2:** OSL dating results (see supplementary material for more details).

Sample	XU and depth	Age (ka)
UJS3	005 (0.45 m)	5.836 ± 0.398
UJS2	012 (0.94 m)	8.701 ± 0.602
UJS1	013 (1.04 m)	9.807 ± 0.665

**Table 3:** AMS radiocarbon ages from the excavation conducted in the hyena den as well as from elsewhere in Umm Jirsan. Calibrated using Calib (v. 8.20) and IntCal20 [76]. Radiocarbon dating analysis conducted at the SUERC Radiocarbon Laboratory, University of Glasgow. Specimens with an asterisk (*) were obtained by Pint [36] and the remainder by Stewart et al. [40].

Lab code	Material	AMS date (bp)	Cal. BP 95.4% (2 σ)
GdA-1156*	Bone (*Homo sapiens*)	150 ± 30	0–46 (19.9%) 56–119 (24.5%) 125–154 (10.9%) 168–232 (27.8%) 238–283 (17.0%)
GdA-1158*	Bone (*Homo sapiens*)	3410 ± 30	3568–3721 (95.0%) 3797–3721 (5.0%)
GdA-1157*	Bone (*Homo sapiens*)	4040 ± 30	4419–4581 (97.6%) 4599–4612 (1.9%) 4772–4778 (0.5%)
GdA-1159*	Bone (Ungulate)	2285 ± 30	2157–2172 (3.2%) 2174–2240 (38.1%) 2300–2351 (58.7%)
GU56831	Bone (*Homo sapiens*)	136 ± 29	8–152 (64.6%) 172–177 (1.2%) 184–202 (5.7%) 206–278 (28.5%)
GU56833	Bone (*Homo sapiens*)	2898 ± 29	2952–3084 (81.9%) 3088–3158 (18.1%)
GU56834	Bone (*Homo sapiens*)	3911 ± 29	4246–4279 (10.6%) 4284–4418 (89.4%)
GU55752	Dentin (*Equus sp*.)	383 ± 28	319–379 (33.2%) 386–390 (0.6%) 426–504 (66.2%)
GU56832	Bone (*Homo sapiens*)	6001 ± 29	6747–6769 (6.5%) 6777–6905 (87.7%) 6908–6936 (5.7%)
GU55753	Dentin (*Equus sp*.)	373 ± 28	318–393 (42.0%) 425–499 (58.0%)
GU55754	Dentin (*Equus sp*.)	4128 ± 32	4529–4562 (7.8%) 4565–4728 (63.3%) 4749–4819 (28.9%)
GU55755	Dentin (*Bos sp*.)	2824 ± 31	2849–3005 (97.5%) 3017–3030 (1.3%) 3045–3056 (1.2%)
GU55756	Dentin (*Equus sp*.)	2532 ± 28	2495–2597 (49.5%) 2616–2643 (17.4%) 2686–2741 (33.1%)
GU56835	Dentin (*Gazella* sp.)	3919 ± 29	4245––4421 (100%)
GU56836	Dentin (*Gazella* sp.)	3551 ± 29	3722–3798 (30.6%) 3819–3924 (67.5%) 3949–3961 (1.9%)
GU55757	Dentin (*Equus sp*.)	2544 ± 31	2496–2596 (42.6%) 2613–2641 (15.6%) 2690–2747 (41.8%)
GU56837	Dentin (*Capra* sp.)	2986 ± 29	3067–3249 (96.1%) 3301–3324 (3.9%)

### Trench 1 lithics and fauna

A total of 44 lithics were recovered from the Trench 1 excavation (Fig 4H–4O), all made of fine-grained dark green obsidian. Thirteen of the lithics were widely distributed in XUs 003 to 010. All remaining 31 lithics (71%) were found in the thin XU 011. Aside from a single core in XU 007 (Fig 4O), which shows bidirectional flaking perhaps by the bipolar method, the lithics from the trench all consist of flakes and chips. The 31 lithics found in XU 011 consist of 13 flakes and 18 chips with an average weight of 2.3 g, although 75% are below 1.3 g. Some flakes have a relatively laminar form, while others are very wide. Overall, the character of the assemblage indicates on site knapping.

A total of 653 faunal bones were recovered (NRSP) from the trench and spoil heap, of which 161 (25% of NRSP) were identifiable to a specific skeletal element (full results of the zooarchaeological and taphonomic analysis are provided in the Supplementary Online Material, S5–S11 Tables in S1 File). Micromammals dominate the assemblage, whereas larger mammals (e.g., equids) and non-mammalian microfauna (e.g., birds, reptiles) are rarely represented. Bone preservation is variable and appears to correlate with animal body size: microfauna show fragmentation and digestion due to predation by raptors but are on the whole comparatively well-preserved, whereas remains of larger animals are fragile and often heavily fragmented.

A minimum number of 15 individuals representing 11 taxa were recovered from the trench and spoil heap (S5 Table in S1 File). Macromammals include small carnivores (NISP = 3, MNI = 1), gazelles (NISP = 2, MNI = 1), caprids (NISP = 2, MNI = 1), and equids (NISP = 2, MNI = 1). Non-mammalian microfauna include birds (NISP = 5, MNI = 1), reptiles (NISP = 4, MNI = 2), and frog (NISP = 1, MNI = 1). Rodents (NISP = 63, MNI = 7) dominate the assemblage and are represented by at least three taxa: *Gerbillus* sp. (gerbil), cf. *Arvicanthis niloticus* (African grass rat), and either *Mus* sp. (mouse) or *Acoyms* sp. (spiny mouse) (S6 Fig in S1 File). Interestingly, throughout the sedimentary sequence there is an inverse relationship between microfauna and larger animals (S7 Fig and S9 Table in S1 File). This suggests that raptors were utilizing the Area C entrance as a roosting site when humans were absent.

Subaerial weathering appears to have had little impact on the assemblage (S7 Table in S1 File), suggesting that bones were buried relatively quickly or, in the case of microfauna, protected from the elements by the pellets in which they were encased. A small number of bones (2.5% of NISP), as well as plant remains, are burnt (S6 Fig in S1 File). Three of these were found in the spoil heap and therefore might be of more recent origin, while a single burnt small animal midshaft fragment was recovered from XU 011. A few small-sized animal midshafts exhibit flake scars and pits that might be of anthropogenic origin, and several bone flakes were also recovered. Several long bones exhibit oblique, curved, and smooth fracture patterns indicative of fracturing while fresh (S10 Table in S1 File). Gastric corrosion is common among micromammal remains. For limb bone elements, corrosion is light and restricted mostly to the epiphyses and protuberances (e.g., trochanters). Most teeth had no signs of digestion (*n* = 6), while the remainder exhibited light (*n* = 2) to moderate (*n* = 2) levels of digestion. Only the mandible bodies were preserved, falling into the low and moderate breakage categories of Fernández-Jalvo et al. [60].

### Stable carbon (δ^13^C) and nitrogen (δ^15^N) isotopes of faunal remains

**Fig 5:**
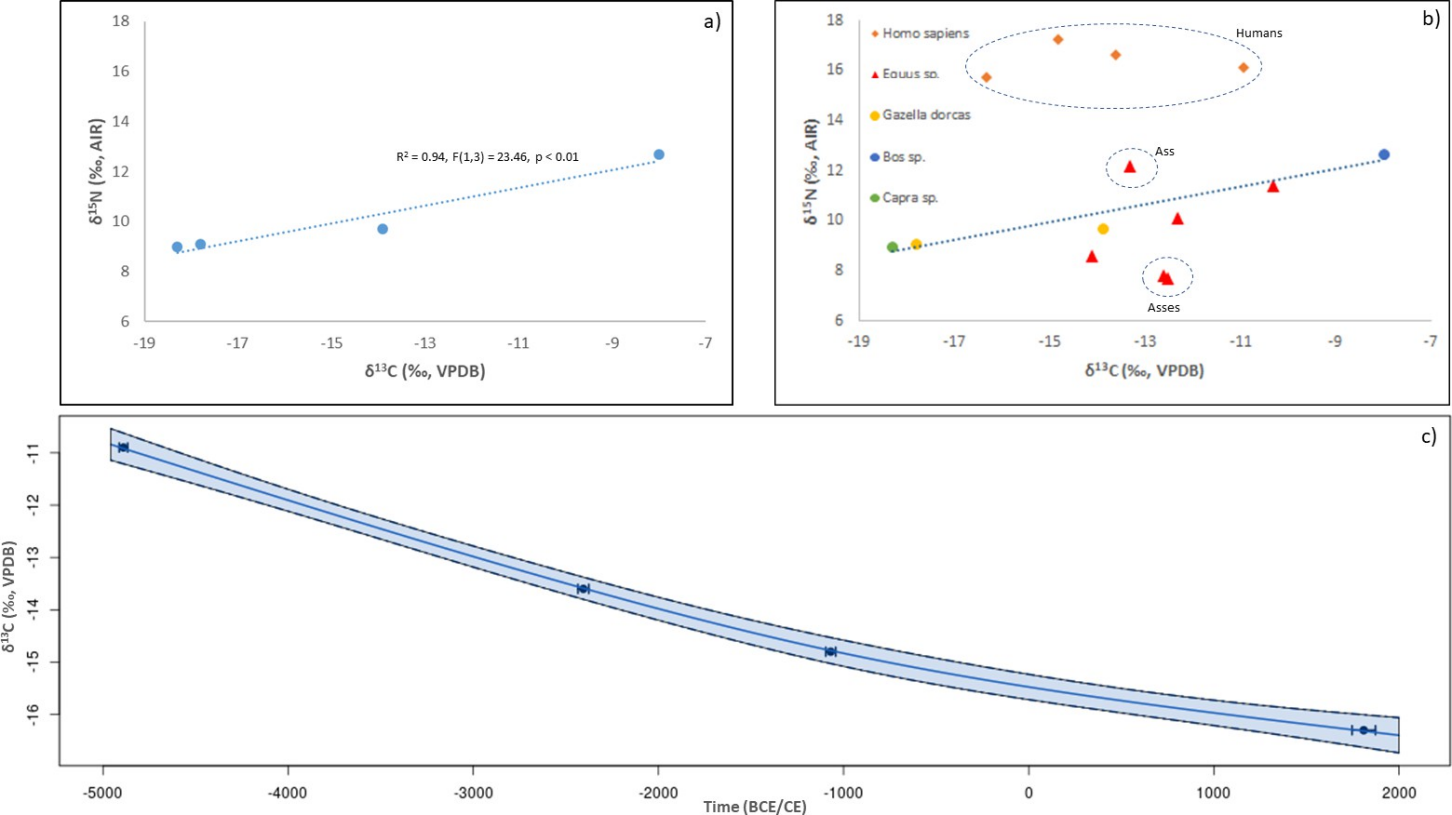
Results of the stable carbon (δ^13^C) and nitrogen (δ^15^N) isotope analyses. (A) Correlation between δ^13^C and δ^15^N isotope ratios for tooth collagen from Umm Jirsan herbivores other than asses. (B) Biplot of δ^13^C and δ^15^N isotope ratios for bone and tooth collagen samples for humans and all herbivores from Umm Jirsan. Ellipses identify human group and groups of asses that do not follow the overall herbivore trend. (C) Bayesian plot of the relationship between chronology and human bone collagen δ^13^C values. The continuous line is the estimated mean while the band represent the 95% credible interval of the standard error of the mean. Calibrated human radiocarbon ages are summarized by the mean (points) and 95% credible intervals (whiskers).

A significant correlation (R^2^ = 0.94, F(1,3) = 23.46, p<0.01) between δ^13^C and δ^15^N values for herbivores (excluding asses) was observed at Umm Jirsan (Fig 5A). Although caution is warranted given the limited dataset (n = 4), the correlation suggests that both domesticates and non-domesticates exploited throughout their movements locally available wild grasses instead of being fed with crops managed by humans. There is a wide range in δ^13^C herbivore values, from -18.3 to -8.0‰, with corresponding Bayesian estimates for the consumption of C_4_ plants ranging from 9±9 to 89±8%. Comparison of the regression trend for grazing herbivores with equid isotopic values shows that three of the latter deviate from the trend (Fig 5B). Two asses, dating to the 15^th^ to 17^th^ centuries CE, exhibited a substantial consumption of C_4_ plants, yet the lowest δ^15^N values of all herbivores. This suggests they were likely fed with plants grown under wetter conditions, either locally available or from other regions. Another equid showed similar δ^13^C values to the previous two, yet higher δ^15^N values, which implies the consumption of plants from manured plots and/or from more arid conditions. No correlation was observed between grazer δ^13^C values and their median calibrated radiocarbon age (R^2^ = 0.12, F(1,3) = 0.4218 p = 0.5623).

Human isotopic results show a wide range in δ^13^C values (-16.3 to -10.9‰) and relatively high and constrained δ^15^N values (15.7 to 17.2‰) (Fig 5B). Bayesian estimates of C_4_ intake by humans vary from 21±14 to 65±10%. This could be accounted for by direct consumption of C_4_ plants, including cereals such as millet or sorghum, or by the consumption of animals fed with C_4_ plants, as observed for Umm Jirsan. Due to a lack of reference plant isotopic values, estimates for the intake of plant versus animal protein are difficult to determine. Nonetheless, the difference of c. 6‰ between the δ^15^N herbivore average (10.3±1.6‰, excluding equids) and the δ^15^N human average (16.4±1.6‰) indicates a heavy intake of animal protein. Under a scenario where we assume a standard δ^15^N offset of 3.5‰ between animal bone collagen and plant protein [77], Bayesian estimates of the dietary protein contribution from animal products range closely from 74±28 to 88±18%. The wide variability in δ^13^C values is indicative of the different individuals consuming different proportions of C_3_ plants. Notably, there is a significant correlation between the median chronological range for each human and respective δ^13^C values (Fig 5C). This shows that, although a tradition for high protein intake was maintained across time, there was a temporal increase in the consumption of animals fed on C3 plants and possibly also some direct consumption of C_3_ plants such as fruits (e.g., dates, figs) or cereals (e.g., wheat) associated with oasis agriculture. While the human and faunal isotopic trends are intriguing, more samples should be sought to further test these correlations.

### Rock art and close environs

Surveys in the area surrounding Umm Jirsan resulted in the discovery of additional archaeological findings (Fig 1). Approximately 2.5 km to the north and 3 km to the west of Umm Jirsan, various subcircular cell-like structures were identified, as well as low densities of lithics including small obsidian flakes. Near the circular structures north of Umm Jirsan a ‘bow-tie’ shaped stone structure, low in height but covering a large area was also recorded. The age of these structures remains unknown.

**Fig 6:**
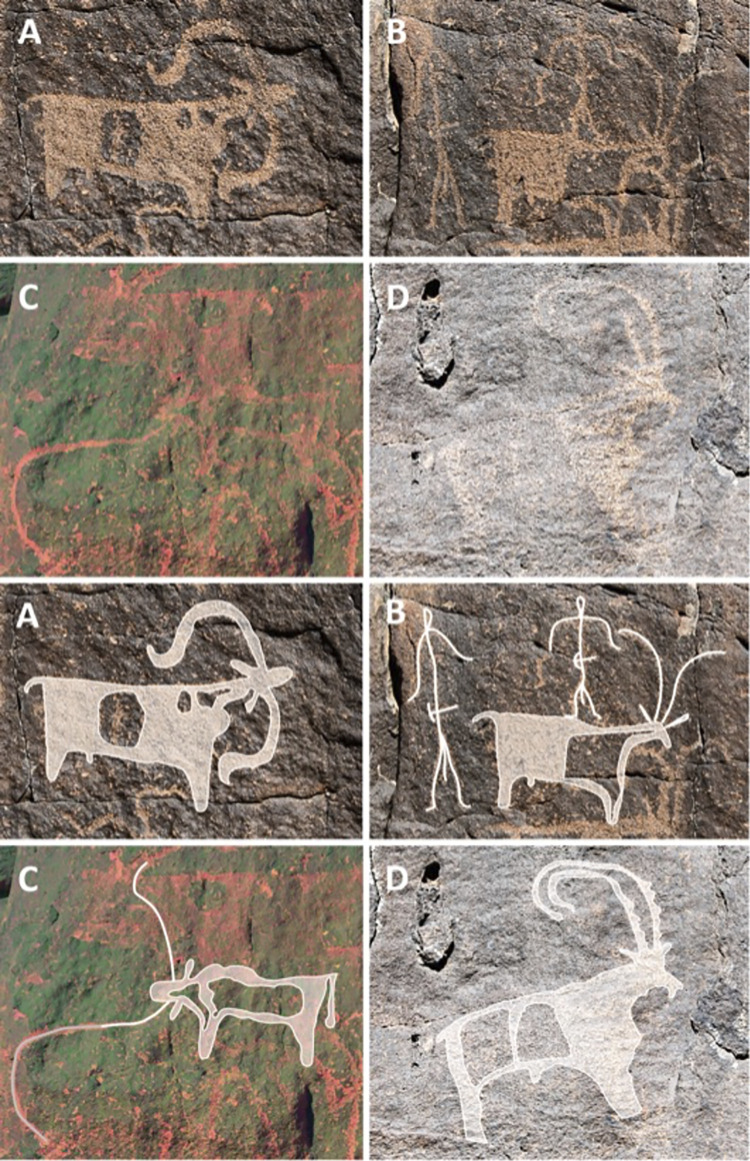
Species identifiable in the rock art of Umm Jirsan. (A) sheep (Panel 8); (B) goat and two stick figures with tools on their belts (Panel 8); (C) long-horned cattle (Panel 6), photo enhanced using the ybk setting on DStretch; (D) ibex with ribbed horns and coat markings (Panel 4). Bottom: tracings of examples A-D.

**Fig 7:**
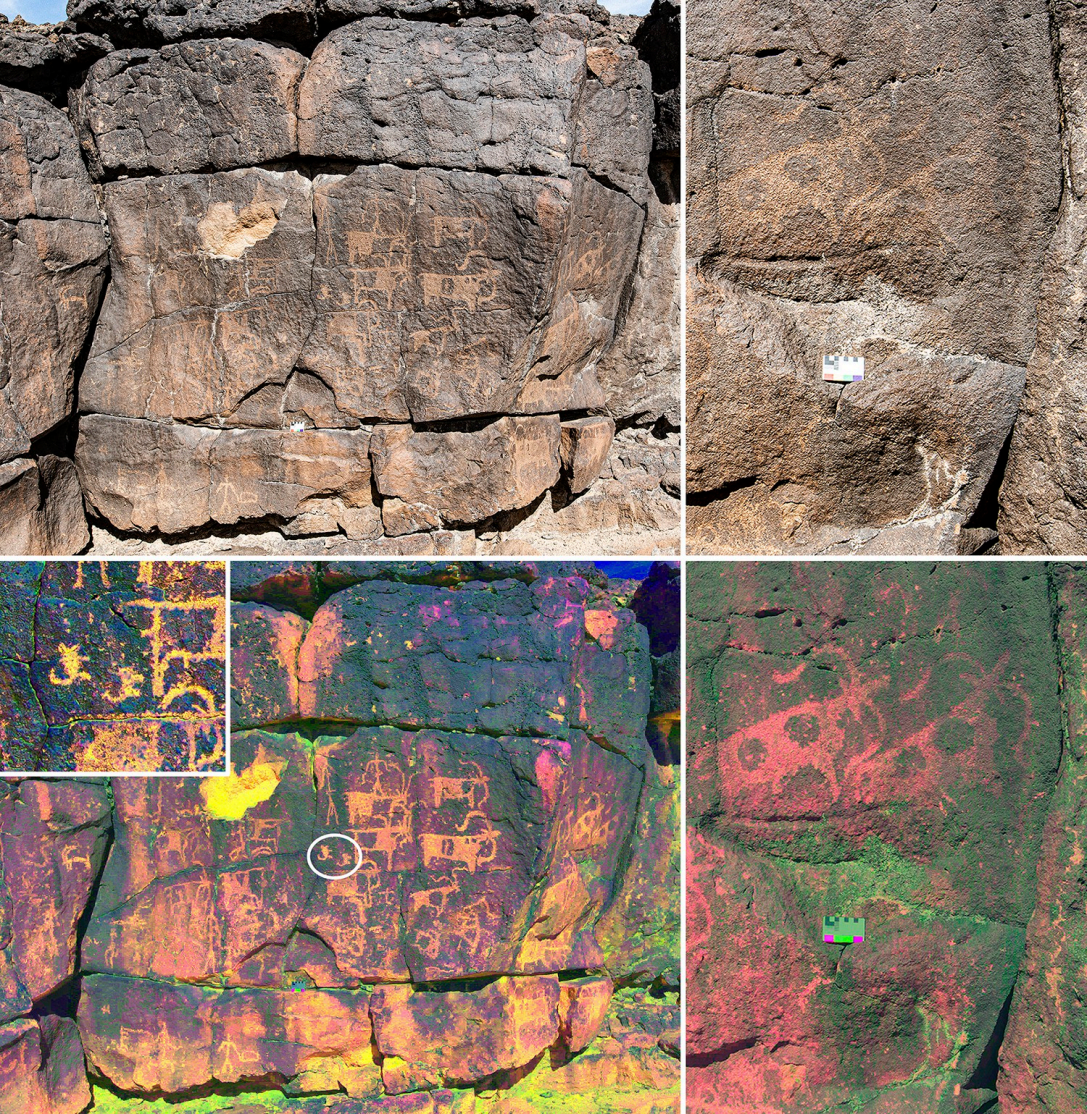
Rock art recorded at Umm Jirsan. Top left: Panel 8, showing a mixed herd of sheep and goats as well as an ibex and several human figures. Bottom left: Photo of Panel 8 modified using the ybk setting on DStretch. Two engravings of dogs are shown in the inset and indicated with a circle on the panel. Both dogs are extremely simplified but show the characteristic curled up tail. Top right: close up of Panel 9, highlighting the difference in rock varnish, likely caused by water running down the rock face. Bottom right: close up of Panel 9 enhanced using the ybk setting on DStretch.

The most interesting locality is a large collapse entrance to another lava tube 1.3 km to the east of Umm Jirsan. The lava tube entrance at the end of the collapse is large, but sediments reach the ceiling after ca. 75 metres. Several small structures (including two small cairns, small clearings, and cells) are located both in the entrance and in the base of the collapse area. Low density non-diagnostic lithics were identified at the base of the collapse area. In addition, 16 rock art panels were documented within the collapse, representing the only documented rock art in the vicinity of Umm Jirsan (Figs 6 and 7). The depicted content is similar across all panels, and most motifs are shown in a similar style. Almost all figures have been produced with very small and fine peck marks that remain visible, attesting to the preservation of the rock surfaces at the site. These peck marks also suggest that a similar tool was used for most of the engravings.

The varnish of the engravings is advanced but not complete. On most panels, engraved lines are distinguishable as a reddish grey colour against the dark grey surface of the varnished rock (Fig 6). However, many of the figures have areas that have darker varnish, and it appears that to an extent this may be the result of vertical streaking, caused by water running down the rock face, which can accelerate or slow varnish formation. The incomplete varnish on surfaces unaffected by steaking is comparable to possible Chalcolithic panels that have been observed at multiple locations in northern Saudi Arabia, although a difference in geological substrate and local environment may result in difference varnish accumulation rates. Stylistically the rock art shares similarities with the Neolithic rock art of northern Arabia. However, there is some indication of time depth, suggesting that the art may cover multiple cultural periods. In some instances, engravings overlap others, such as a human figure superimposed over an ibex on panel 7, or a bovid superimposed over a cow/bull on panel 6 (Fig 6C). Nevertheless, these superimpositions could have been achieved within a relatively short time span. Only two substantially later additions can be observed, both carved in a more simplistic style, and coated with markedly lighter varnish: a stick-figure ibex on the upper right of panel 7, and a stick-figure human on the lower left of panel 8. These two figures are also pecked with a cruder tool, producing larger and less precisely placed peck marks.

Based on stylistic conventions, four different bovid depictions can be identified—cattle, sheep, goat, and possibly ibex (Fig 6). Goats are depicted with their horns rising in almost parallel lines, with the tips curved outwards (Fig 6B). Another type of bovid is shown with the horns curving back in large arches. In some cases, the ridges that are typical for ibex horns can be identified (Fig 6D) and it is possible that bovids with large arched horns represent ibex. Sheep are depicted with their horns curving back over the neck and ears in a characteristic curve (Fig 6A). Several bovids with backward curving horns are shown with very large, exaggerated horns, giving them a cattle-like appearance (Fig 6). However, the direction and shape of the horns, combined with a very short tail suggest these are all sheep. Only two bovid depictions could be confidently identified as cattle, and both have large forward pointing horns. In one of the depictions the tail is preserved and is depicted as long and downward pointing as is typical for cattle (Fig 6C).

Following this stylistic distinction, sheep are the most commonly represented animal in the rock art, with 23 identifiable depictions. In addition, 15 ibex, seven goat, and two cattle were identified and these are frequently associated with human figures in apparent herding scenes (S12 Table in S1 File). The inclusion of ibex-like animals, in mixed scenes with domesticated species is interesting. However, the possibility remains that some of the bovids with backward arching horns represent a different type of goat breed rather than ibex. Panel 2 shows an animal with a mixture of both traits with backward curving horns which flare outwards at the tip.

At least six herding scenes were identified on panels 2, 7, 8 (2 scenes), and 9. This is notable as these are generally quite rare in the rock art of northern Arabia. At least one of the pastoral scenes also shows the use of dogs (panel 8) to herd a mixed herd of goats and sheep (Fig 7, bottom left and inset). The human figures are very simple, most are stick-figure like with straight legs and curved arms, which echo the horn shape of the sheep depictions (Fig 6B).

Given the content and advanced varnish, the engravings most likely relate to the later occupation phase of the cave, perhaps during the middle Holocene. The dominance of sheep and goat is significant, as they are rarely depicted in the rock art of northern Arabia, particularly in oasis settings such as Jubbah and AlUla [6], as well as sites such as Shuwaymis [13] which are dominated by cattle.

Finally, we surveyed the volcanic area around Jebel Abyad (Fig 1) to attempt to locate the source(s) of obsidian found at and around Umm Jirsan (S4 Fig in S1 File). At two localities (named Locality 1 and 2), obsidian clasts and lithics were abundant, which, at the former, included Levallois lithics as well as probable Holocene structures. At a third locality (Locality 3), lithic artefacts were also identified but unlike the other two localities which had a rather mixed character, this locality appears to be comprised entirely of Middle Palaeolithic artefacts. Cores showed a focus on centripetal preparation, while most of the larger Levallois flakes have primarily unidirectional scar patterns, often with additional minor lateral or distal preparation. We also collected some obsidian blocks for knapping experiments. The obsidian was of reasonable quality for flaking, but is very brittle. The prevalence of edge damage on the experimental sample matched that of the archaeological materials observed at Umm Jirsan. Images of archaeological and our experimentally knapped obsidian are shown in S5 Fig in S1 File.

## Discussion

The excavation and survey work conducted in and around Umm Jirsan reveal repeated occupations of the lava tube and surrounding region by Chalcolithic/Bronze Age, Neolithic, and possibly pre-Neolithic peoples over at least the past seven thousand years and perhaps for as long as ten thousand years. The occupation at Umm Jirsan represents one of the first documented archaeological cave sites in the Arabian interior, and one of the few sites in Saudi Arabia dated to the early to mid-Holocene. The site highlights the potential for archaeological surveys and excavations in underground cave settings in the region for the recovery of novel natural and cultural archives.

Based on the radiocarbon and OSL dating, the age of the main occupation layer (XU 011) falls between ca. 10,300 and 7,000 years BP. This age range overlaps with other early Holocene archaeological sites in northern Arabia, notably Al Rabyah, Jebel Qattar, Jebel Oraf, and Sahout, where distinctive Epipalaeolithic/PPN artefacts in the form of El-Khiam points and Helwan points and bladelets hint at cultural connections to the Levant [7, 10, 12]. However, the lithic assemblages recovered from the Trench 1 excavation and surrounding area lack diagnostic forms such as arrowheads or geometric microliths which would allow comparisons with Epipalaeolithic/Neolithic assemblages in the wider region. A Neolithic presence at Umm Jirsan is attested by the recent identification of a human cranium dated to ca. 6,800 years BP from Area A, the hyena accumulation at the western end of the lava tube [40]. This age matches well with recent radiocarbon dates obtained from mustatils in the Harrat Khaybar, as well as circular domestic structures from the nearby Harrat ‘Uwayrid, which suggest that the construction and use of these megalithic structures largely took place between ca. 7,200 and 6,800 years BP [16, 17, 20, 21], with the age of this particular individual perhaps related to the final stages of the use of these structures.

Later occupations are evident at Umm Jirsan, as documented by the Area D surface lithic scatter and the nearby rock art which likely dates to the Chalcolithic, as well as the four human cranial remains recovered from elsewhere at Umm Jirsan dating to between ca. 4,500 and 3,000 years BP [36, 40]. There is again an interesting correspondence between the age of the archaeological findings at Umm Jirsan and megalithic structures in the region, in this case the pendant tombs, dating to between ca. 4,600 and 4,000 years BP [18, 31]. These structures form what have been termed ‘funerary avenues’ [31]. Essentially, these avenues comprise composite path and monumental features connecting oases and, in some instances, extending over hundreds of kilometres. Notably, Umm Jirsan is situated between the Khaybar oasis on the one hand, and al Ha’it and al Huwayyit oases on the other, each of which is connected by extended funerary avenues (see Fig 1).

If, as Dalton and colleagues [31] suggest, these avenues were used by pastoralists during the movement of their herds between oases and hinterland pastures, it seems probable that Umm Jirsan marks a stopping off point. Clearly, pastoralists visited the area, as evidenced by probable Chalcolithic depictions of goat, sheep, and cattle herding scenes in the rock art and the recovery of livestock remains. For such pastoralists, Umm Jirsan may have served as a place of refuge, providing protection from the sun and wind and potentially as a natural reservoir of water. Indeed, the use of lava tubes as water reservoirs in antiquity has been suggested previously. In the Harrat al-Sham (Black Desert) of eastern Jordan, artificial channels leading to and damming walls within lava tubes have been documented, as well as various structures (e.g., walls, crescent-shaped shelters, and possible “post holes”) clearly associated with the lava tube entrances [78–81]. Although the age and function of these various features is not yet known, some scholars have suggested that they date to prehistory [81], and it is of note that the Late Chalcolithic/Bronze Age settlement of Jawa is nearby to these lava tubes. Even today, some of these caves are used as sheep pens [80].

Additional insights regarding human herding and dietary practices over time can be gleaned from the isotopic data. Human isotopic results show a wide range in δ^13^C values and relatively high and constrained δ^15^N values. The wide variability in the former indicates that individuals consumed different proportions of C_3_ plants and/or C_3_-consuming animals, while estimates of the dietary protein contribution from animal products range closely from 74–88%. Notably, there is a significant correlation between the median chronological age of each of the human remains and their respective δ^13^C values. Taken together, this suggests a tradition of high protein alongside an increase over time in the consumption of C_3_ plants (e.g., fruits, cereals) and/or browsers (e.g., goats), with the Neolithic-aged individual having the lowest proportion of C_3_ resources in their diet. The increasing trend in C_3_ resource consumption may relate to the arrival of Bronze Age oasis agriculture which saw the emergence of sophisticated farming and water management technologies that allowed people to cultivate an increasingly diverse array of plant foods, as well as settle more permanently in the deserts [2, 82]. This is well documented in the Tayma Oasis some 200 km northwest of Khaybar, where palynological evidence indicates the cultivation of grapevine (*Vitis vinifera*) and fig (*Ficus*) by as early as ca. 5,300 years BP and the farming of date palms (*Phoenix*) before 3,000 years BP [83]. Although no direct evidence for early agriculture exists yet for Harrat Khaybar, the oasis, with its large catchment area, deep sediments and wadi channels, underlying geology, and shallow water table [18, 84, 85], would have been amenable to oasis agriculture during Bronze Age times.

The strong correlation between faunal δ^13^C and δ^15^N suggests that these animals consumed grasses locally available instead of being fed with crops managed by humans. For the latter, crop management practices such as irrigation or manuring would likely deviate isotopic values from the observed correlation. Interestingly, the δ^13^C values indicate highly varied diets, with some individual consuming mostly C_4_ plants, whereas others incorporated into their diets a significant proportion C_3_ and/or CAM photosynthesizing plants (e.g., as sedges and succulents). Only the more recent 15^th^ to 17^th^ century CE equids remains show a deviation from this trend, with δ^13^C values that suggest they were fed with plants grown under wetter conditions, either locally available or from other regions.

Given the limited size of our dataset and lack of mobility isotopic data (e.g., O or Sr isotopes) it is difficult to put forward robust interpretations on animal mobility. It is possible that the herbivores were fed in two distinct ecozones having extreme isotopic end-members. In this case, one would expect the observed correlation between δ^13^C and δ^15^N for grazer bone collagen to result from different residence times and seasonal mobility across end-members. However, it is also possible that there were an unknown number of ecozones with intermediate isotopic values spread across extreme isotopic values. More data is needed to test these hypotheses.

The rock art nearby Umm Jirsan attests to prehistoric livestock practices and herd composition in the region. Of particular note is the dominance in the rock art of sheep and goat, which are rarely depicted in other oasis settings in northern Arabia, such as at Jubbah, or at sites like Shuwaymis [13], which are dominated by cattle. It is possible that the local environment made sheep and goat herding more economically, and perhaps more symbolically, important. Hunting of gazelle and perhaps of ibex is also attested at the site in the rock art, consistent with the faunal remains from Trench 1.

Both domestic (e.g., caprids, cattle) and wild (e.g., gazelles) animals were processed and/or consumed at the site, with evidence pointing to people as the likely accumulators of these remains for several reasons. Firstly, the large open entrance to the eastern passage is not an ideal denning spot for carnivores like striped hyena and foxes, which tend to prefer more sheltered settings [52]. Secondly, the assemblage is rather distinct from clearly carnivore accumulated assemblages found elsewhere throughout Umm Jirsan. This includes the large hyena accumulated caches as reported in [40], as well as small fox accumulated caches which comprise primarily small accumulations of microfauna (e.g., rodents, birds) remains surrounding small cracks and crevices in the cave walls. Thirdly, the inverse relationship between the micro and macrofauna is easily explainable by a human presence—through the disturbance of raptors via activities such as hunting and fire use—but less so by a carnivore presence. And lastly, the faunal remains are associated with lithic artefacts, while some of the bones have possible evidence for human butchery in the form of burning, percussion pits, and fresh fracturing (i.e., midshafts with oblique, curved, and smooth fracture characteristics). Taken together, the evidence points to humans as the main accumulating agent of the macromammal remains recovered from the excavation.

In contrast, the microfauna found at the site appear to be the result of raptor predation, most likely some nocturnal owl based on the degree of breakage and digestive corrosion. There is a clear inverse relationship between the micro and macrofauna throughout the sequence. Namely, when larger fauna are present, microfauna are largely absent, and *vice versa*, indicating that raptors were using the cave while people were absent. A simple reading of S6 Fig in S1 File suggests perhaps as many as four distinct occupational phases of the cave by people, possibly related to the various Neolithic and Chalcolithic/Bronze Age occupations of the area. While little is currently known on questions such as occupation continuity in the area, our findings suggest pulses of occupation reflected in both the cultural and faunal records.

## Conclusions

Our findings point to use of Umm Jirsan by predominantly pastoral populations that had increasing links through time with oasis settlements. The lava tube does not appear to have served as a permanent habitation location, but rather as a site that likely lay on herding routes and that allowed access to shade and water for passing herders and their animals. Prior to this, as well as during pastoral periods, the lava tube was likely also linked with hunting activities, which probably remained a cornerstone of local economies into the Bronze Age. Lava tubes like Umm Jirsan offered a rich resource for human populations living in arid habitats and provide important insights into the resources these populations drew upon to increase their resilience in a challenging environment.

Our research at Umm Jirsan highlights the value of further research and testing of northern Saudi Arabia’s rich record of caves and rock shelters. These records hold substantial potential to address major limitations of the current archaeological record, which relates to open air sites subjected to major post-depositional transformations and taphonomic degradation. Future research should target additional sites, drawing upon the full potential of multiproxy methods to document linked cultural and environmental records, in a key and fascinating arid region of the Eurasian continent.

## Supporting information

S1 File Supplementary online material. Additional information on the OSL dating, stable carbon and nitrogen isotope analyses, and archaeological findings.
(DOCX)
