# Supplementary material for: First evidence for human occupation of a lava tube in Arabia: The archaeology of Umm Jirsan Cave and its surroundings, northern Saudi Arabia
Source: PLoS One. 2024 Apr 17;19(4):e0299292. doi: 10.1371/journal.pone.0299292 (PMC11023468; doi:10.1371/journal.pone.0299292)
Supplement: S1 File — Additional information on the OSL dating, stable carbon and nitrogen isotope analyses, and archaeological findings. (DOCX) [file pone.0299292.s001.docx]

**First evidence for human occupation of a lava tube in Arabia: the archaeology of Umm Jirsan Cave and its surroundings, northern Saudi Arabia**

**Supplementary Online Material**

# Optically Stimulated Luminescence (OSL) dating

## Methods

*Equipment*

Luminescence measurements were performed using a Risø TL/OSL-DA-20. Stimulation of multi-grain aliquots was carried out using an infrared (875 nm) LED array (nominal power density 135 mW/cm^2^). IRSL feldspar luminescence emissions were detected through a combination of 2 mm Schott BG-39 and 3 mm Schott BG-3 filters. Luminescence signals were measured using an Electron Tubes Ltd 9235QB photomultiplier tube. All irradiations were performed using a 1.48 GBq ^90^Sr/^90^Y beta source, calibrated relative to the National physical Laboratory, Teddington ^60^Co gamma-source (Hotspot 800; Armitage and Bailey, 2005).

*Equivalent dose determination*

The Umm Jirsan sediments comprise feldspars rich silts originating from the multiple eruptions of the nearby Khaybar volcanos. The feldspars extracted from the samples were analysed following a pIR-IRSL protocol (Buylaert et al., 2012; Li et al., 2014; Thiel et al., 2011). Preheat temperatures were selected following a preheat plateau test; a 60 s 250°C preheat was used followed by pIR-IRSL measurements at 50°C and 225°C (see Table S1).

Regeneration doses were chosen to bracket the expected palaeodoses. The dose response curves were fitted with a saturating exponential, or saturating exponential plus linear function, chosen for each individual curve on the basis of goodness of fit. Curve fitting, D_e_ determination and Monte Carlo simulation were performed using version 4.31.9 of the Luminescence Analyst software (Duller, 2007).

A dose recovery and residual tests were conducted to test the applicability of our pIR-IRSL SAR protocol. Sixteen aliquots were bleached during six hours in a solar irradiator. Eight of these were given a known dose of 28.75 Gy, which was recovered with a 1.03 ± 0.02 Gy ratio. The remaining eight were tested for residual dose. The residual dose estimated using the pIR-IRSL protocol was 0.45 ± 0.21 Gy. This residual dose was subtracted from the palaeodoses.

Net pIR-IR_225_ and signals were calculated by subtracting a mean background (calculated from the signal observed during the last 60s of stimulation) from the total signal emitted over the first 2 s (Buylaert et al., 2013). Fading rate was measured on 12 aliquots for each sample following Huntley and Lamothe (2001) and Auclair et al. (2003) using Analyst. These yielded low g-values for the pIR-IR225 signals, 0.91 ± 0.31%, 1.11 ± 0.22% and 0.89 ± 0.17% respectively for UJS1, 2 and 3. Since these values may indicate the absence of fading (Buylaert et al., 2012; Lowick et al., 2012; Roberts, 2012) the samples were not corrected for fading.

*Burial dose determination (D_b_)*

The statistical models most frequently used to extract a single burial dose (D_b_) from the distribution of measured D_e_ values are the central age model (CAM; Galbraith et al., 1999; Roberts et al., 2000) and the Minimum Age Model (MAM; Galbraith and Laslett, 1993). CAM is designed for well-bleached samples which have experienced no post-depositional mixing. This model calculates D_b_ from a weighted mean of the measured D_e_s. The MAM is used when the sample contains both fully and partially bleached grains. MAM fits a truncated normal distribution to log D_e_ values, with the truncation point giving D_b_.

After the analysis of the degree of skewness, the kurtosis and the overdispersion of the D_e_ distributions following Bailey and Arnold (2006), CAM was considered the most appropriate statistical model to use with our UJS1 and UJS3 datasets while MAM was used for UJS2.

*Dose rate determination*

External dose rates for the samples were calculated from the concentrations of potassium, thorium, and uranium measured via ICP-MS analysis (Activation Laboratories, Ltd., Canada) using absorption factors from Brennan (2003) and conversion factors from Adamiec and Aitken (1998). The absorbed dose due to rubidium was taken from Readhead (2002). K-feldspars internal dose rate was calculated assuming grains contain 12.5 ± 0.5% potassium (Huntley and Baril, 1997) and 400 ± 100 ppm rubidium-87 (Huntley and Hancock, 2001).

Dose rates were corrected for: (i) alpha efficiency (0.15 ± 0.05 for sand-sized feldspars; Balescu and Lamonthe (1994); (ii) attenuation due to grain size feldspars (Brennan, 2003); and (iii) moisture content (Aitken, 1985). A mean burial water content of 10 ± 5 % was assumed for all samples. The 2σ uncertainty on water content encompasses completely dry conditions (0%) and saturation for 25% of the burial period (20%), representing the full range of reasonable mean water content scenarios for these samples.

Cosmic dose rates were calculated using site location, sediment overburden, and present-day burial depths (Prescott and Hutton, 1994).

## Results

**Table S1.** The pIR-IR Single-Aliquot Regenerative-dose protocol used in this study.

| **Step** | **Feldspar pIR-IR_225_** |
| --- | --- |
| 1 | Give regenerative dose^1^ |
| 2 | Preheat 1 (250 ⁰C for 60 s) |
| 3 | IR diode stimulation (50 ⁰C for 200 s) |
| 4 | IR diode stimulation (225 ⁰C for 200 s) |
| 5 | Give test dose |
| 6 | Preheat 2 (250 ⁰C for 60 s) |
| 7 | IR diode stimulation (50 ⁰C for 200 s) |
| 8 | IR diode stimulation (225 ⁰C for 200 s) |
| 9 | IR diode stimulation (325 ⁰C for 100 s) |
| 10 | Return to step 1 |

^1^In the first cycle, where the natural luminescence intensity is observed, no regenerative dose was administered.

**Table S2.** External dose rates.

| Sample | Alpha dose rate (Gy/ka) | Beta dose rate (Gy/ka) | Gamma dose rate (Gy/ka) | Internal dose rate (Gy/ka) | Cosmic dose rate (Gy/ka) | Total dose rate (Gy/ka) |
| --- | --- | --- | --- | --- | --- | --- |
| UJS1 | 0.078 ± 0.01 | 1.43 ± 0.137 | 0.61 ± 0.069 | 0.661 ± 0.105 | 0.209 ± 0.021 | 2.989 ± 0.188 |
| UJS2 | 0.044 ± 0.005 | 1.004 ± 0.099 | 0.539 ± 0.061 | 0.661 ± 0.105 | 0.212 ± 0.021 | 2.459 ± 0.159 |
| UJS3 | 0.051 ± 0.006 | 1.042 ± 0.102 | 0.494 ± 0.055 | 0.661 ± 0.105 | 0.244 ± 0.024 | 2.491 ± 0.159 |

**Table S3.** Palaeodoses and Ages. A residual dose of 0.45 ± 0.21 Gy was subtracted before Db estimations.

| Sample | Total dose rate (Gy/ka) | Db (Gy) | Age (ka) |
| --- | --- | --- | --- |
| UJS1 | 2.989 ± 0.188 | 29.31 ± 0.75 | 9.807 ± 0.665 |
| UJS2 | 2.459 ± 0.159 | 21.4 ± 0.54 | 8.701 ± 0.602 |
| UJS3 | 2.491 ± 0.159 | 14.54 ± 0.35 | 5.836 ± 0.398 |


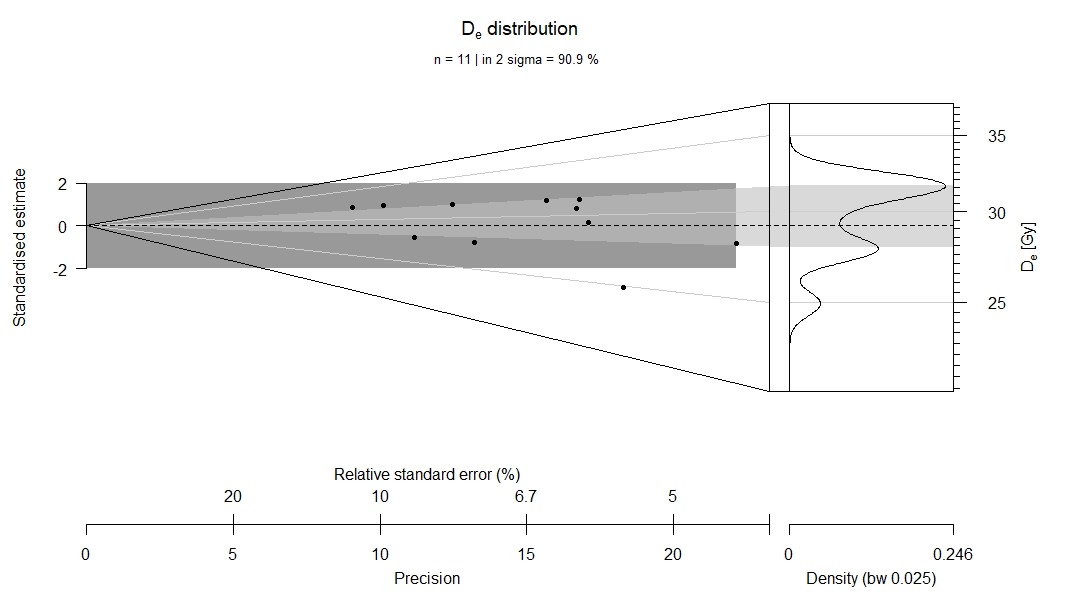


**Figure S1.** Abanico plot UJS1. Data is centred around the CAM Db. The grey shaded areas represent the 2 sigma standardised estimates


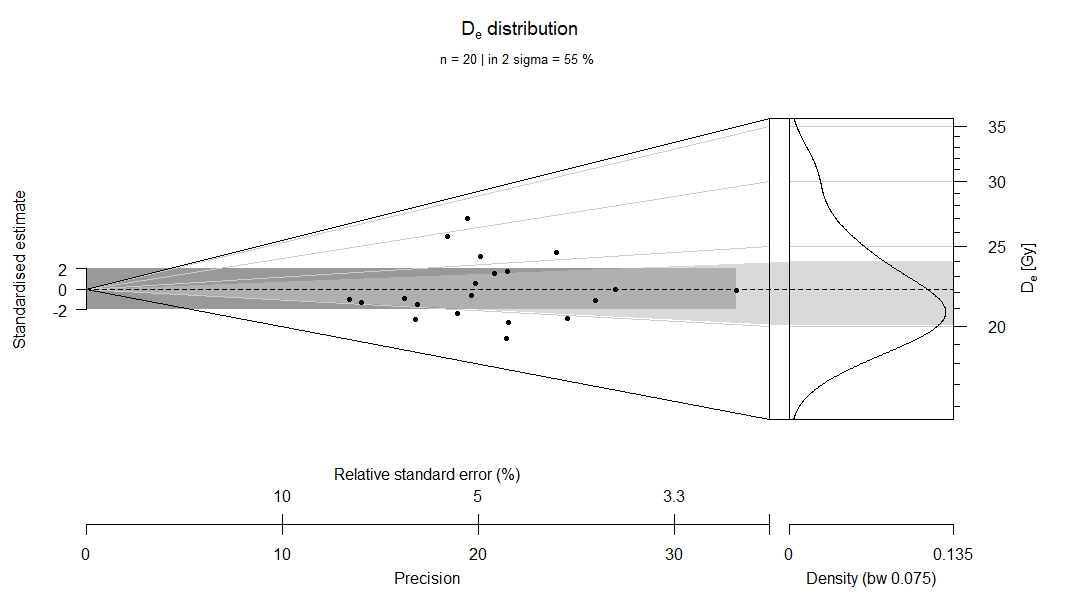


**Figure S2.** Abanico Plot UJS2. Data is centred around the CAM Db. The grey shaded areas represent the 2 sigma standardised estimates.


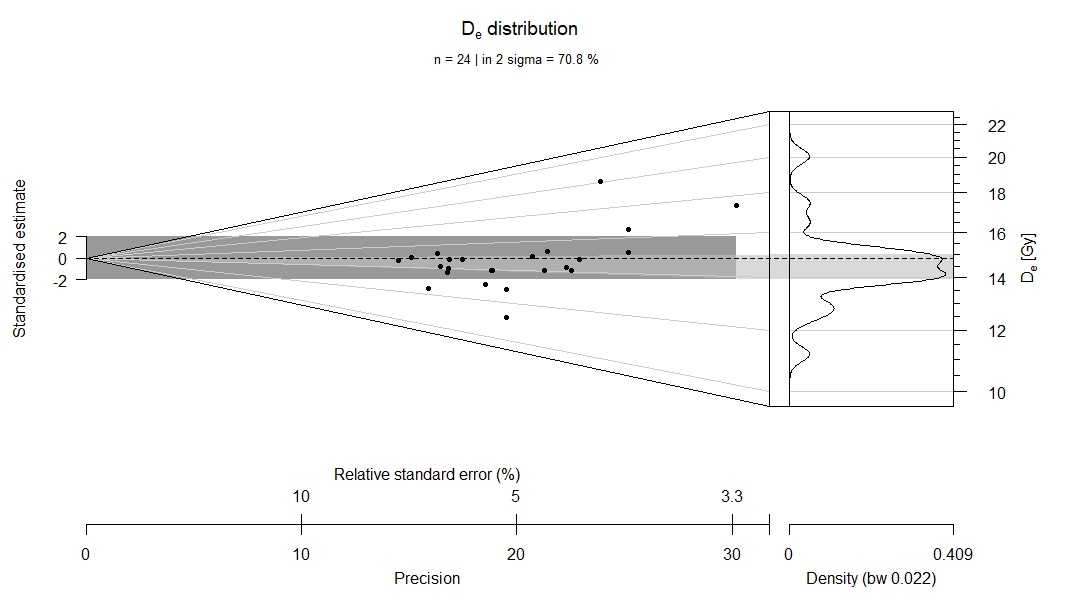


**Figure S3.** Abanico plot UJS3. Data is centred around the CAM Db. The grey shaded areas represent the 2 sigma standardised estimates.

# Stable carbon (δ^13^C) and oxygen (δ^18^O) analysis results

**Table S4.** AMS radiocarbon ages and corresponding stable carbon (δ^13^C), nitrogen (δ^15^N), and atomic C:N results.

| **Lab code** | **Material** | **AMS date (bp**) | **δ^13^C** | **δ^15^N** | **Atomic C:N** |
| --- | --- | --- | --- | --- | --- |
| GU56831 | Bone (*Homo sapiens*) | 136 ± 29 | -16.3 | 15.7 | 3.6 |
| GU56833 | Bone (*Homo sapiens*) | 2898 ± 29 | -14.8 | 17.2 | 3.4 |
| GU56834 | Bone (*Homo sapiens*) | 3911 ± 29 | -13.6 | 16.6 | 3.4 |
| GU56832 | Bone (*Homo sapiens*) | 6001 ± 29 | -10.9 | 16.1 | 3.3 |
| GU55752 | Dentine (*Equus sp.*) | 383 ± 28 | -12.6 | 7.8 | 3.5 |
| GU55756 | Dentine (*Equus sp.*) | 2532 ± 28 | -13.3 | 11.1 | 3.6 |
| GU55757 | Dentine (*Equus sp.*) | 2544 ± 31 | -12.3 | 10.1 | 3.6 |
| GU55753 | Dentine (*Equus sp.*) | 373 ± 28 | -12.5 | 7.7 | 3.5 |
| GU55754 | Dentine (*Equus sp.*) | 4128 ± 32 | -10.3 | 11.4 | 3.7 |
| GU55755 | Dentine (*Bos sp.*) | 2824 ± 31 | -8.0 | 12.7 | 3.5 |
| GU56835 | Dentine (*Gazella* sp.) | 3919 ± 29 | -13.9 | 9.7 | 3.3 |
| GU56836 | Dentine (*Gazella* sp.) | 3551 ± 29 | -17.8 | 9.1 | 3.4 |
| GU56837 | Dentine (*Capra* sp.) | 2986 ± 29 | -18.3 | 9.0 | 3.3 |

# Lithics, fauna, and excavation

**Table S5.** Taxonomic list from the Trench 1 excavation at Umm Jirsan.

| **Class** | **Order** | **Family** | **Taxon** | **Common name** | **NISP** | **MNI** |
| --- | --- | --- | --- | --- | --- | --- |
| **Amphibia** | Anura |  | Gen. et sp. indet. | Frog | 1 | 1 |
| **Reptilia** | Squamata |  | Gen. et sp. indet.  Gen. et sp. indet. | Lizard  Snake | 3  1 | 1  1 |
| **Aves** |  |  | Gen. et sp. indet. | Bird | 5 | 1 |
| **Mammalia** | Rodentia  Artiodactyla  Perissodactyla  Carnivora | Muridae  Bovidae  Equidae  Canidae | cf. *Arvicanthus niloticus*  *Gerbillus* sp.  cf. *Mus / Acomys*  Gen. et sp. indet.  *Gazella* sp.  Caprid sp.  *Equus* sp.  Gen. et sp. indet. | African grass rat  Gerbil  Mouse / spiny mouse  Rodent  Gazelle  Caprid  Equid  Canid | 3  2  14  44  2  2  2  3 | 1  2  4  1  1  1  1 |
| **Total** |  |  |  |  | 82 | 15 |

**Table S6.** Complete skeletal inventory of rodents from the Trench 1 excavation at Umm Jirsan.

|  | **NISP** | **MNE** | **%MNE** |
| --- | --- | --- | --- |
| Crania + maxilla | 2 | 1 | 8 |
| Mandible | 6 | 6 | 50 |
| Isolated incisor | 8 | - | - |
| Vertebra | 1 | 1 | 8 |
| Rib | 0 | 0 | 0 |
| Scapula | 0 | 0 | 0 |
| Pelvis | 2 | 2 | 17 |
| Humerus   - Complete - Proximal epiphysis - Proximal epiphysis + shaft - Midshaft - Distal epiphysis + shaft - Distal epiphysis | 4  1  -  1  -  2  - | 3 | 25 |
| Radius   - Complete - Proximal epiphysis - Proximal epiphysis + shaft - Midshaft - Distal epiphysis + shaft - Distal epiphysis | 2  -  -  -  -  1  - | 2 | 17 |
| Ulna | 0 | 0 | 0 |
| Metacarpal | 0 | 0 | 0 |
| Femur   - Complete - Proximal epiphysis - Proximal epiphysis + shaft - Midshaft - Distal epiphysis + shaft - Distal epiphysis | 12  4  -  4  -  4  - | 12 | 100 |
| Tibia   - Complete - Proximal epiphysis - Proximal epiphysis + shaft - Midshaft - Distal epiphysis + shaft - Distal epiphysis | 10  1  -  3  6  -  - | 10 | 83 |
| Metatarsal | 1 | 1 | 8 |
| Astragalus | 0 | 0 | 0 |
| Calcaneum | 0 | 0 | 0 |
| Carpal/tarsal | 0 | 0 | 0 |
| Sesamoid | 0 | 0 | 0 |
| Phalanx | 5 | 5 | 42 |
| Sacrum | 0 | 0 | 0 |
| Long bone shaft | 10 | - | - |
| Total | 63 | 43 | - |

**Table S7.** Results of the taphonomic analysis from the Trench 1 excavation (Layers 1–6). Excludes small (<20 mm) unidentifiable fragments.

|  | **Spoil heap** | **Layer 1** | **Layer 2** | **Layer 3** | **Layer 4** | **Layer 5** | **Layer 6** |
| --- | --- | --- | --- | --- | --- | --- | --- |
| NRSP | 48 | 3 | 2 | 5 | 89 | 52 | 11 |
| NISP | 19 | 2 | 2 | 5 | 15 | 28 | 1 |
| Weathering  0  1  2  3  4  5 | 16  0  0  0  0  0 | 0  0  0  0  0  0 | 0  0  0  0  0  0 | 5  0  0  0  0  0 | 7  0  0  0  0  0 | 18  0  0  0  0  0 | 0  0  0  0  0  0 |
| Circ. complete  Type 1  Type 2  Type 3 | 2  0  3 | 0  0  0 | 0  0  0 | 0  0  2 | 3  0  0 | 6  0  5 | 0  0  0 |
| Manganese staining | 4 | 0 | 0 | 0 | 0 | 2 | 0 |
| Striae | 1 | 0 | 0 | 0 | 0 | 1 | 0 |
| Gastric corrosion | 2 | 0 | 0 | 0 | 1 | 11 | 0 |
| Burning | 3 | 0 | 0 | 0 | 0 | 0 | 0 |

**Table S7.** *Continued*

|  | **Layer 7** | **Layer 8** | **Layer 9** | **Layer 10** | **Layer 11** | **Layer 13** | **Total** |
| --- | --- | --- | --- | --- | --- | --- | --- |
| NRSP | 7 | 53 | 24 | 144 | 214 | 1 | 653 |
| NISP | 5 | 31 | 23 | 22 | 7 | 1 | 161 |
| Weathering  0  1  2  3  4  5 | 5  0  0  0  0  0 | 21  0  0  0  0  0 | 17  0  0  0  0  0 | 22  0  0  0  0  0 | 4  0  0  0  0  0 | 1  0  0  0  0  0 |  |
| Circ. complete  Type 1  Type 2  Type 3 | 5  0  0 | 10  2  7 | 0  0  11 | 5  1  9 | 0  0  0 | 0  0  0 |  |
| Manganese staining | 0 | 0 | 0 | 0 | 0 | 0 |  |
| Striae | 0 | 0 | 0 | 1 | 0 | 0 |  |
| Gastric corrosion | 0 | 4 | 7 | 6 | 3 | 0 |  |
| Burning | 0 | 0 | 0 | 0 | 1 | 0 |  |

**Table S8.** Specimen length (mm).

|  | **Spoil heap** | **Layer 1** | **Layer 2** | **Layer 3** | **Layer 4** | **Layer 5** | **Layer 6** |
| --- | --- | --- | --- | --- | --- | --- | --- |
| Specimen length (mm)  <20  20–29  30–39  40–49  50–59 | 40  4  4  0  0 | 0  1  1  1  0 | 2  0  0  0  0 | 5  0  0  0  0 | 79  7  2  1  0 | 45  7  0  0  0 | 11  0  0  0  0 |
|  | **Layer 7** | **Layer 8** | **Layer 9** | **Layer 10** | **Layer 11** | **Layer 13** | **Total** |
| Specimen length (mm)  <20  20–29  30–39  40–49  50–59 | 5  1  1  0  0 | 52  1  0  0  0 | 21  2  1  0  0 | 126  8  4  4  2 | 186  21  6  0  1 | 0  1  0  0  0 | 572  53  19  6  3 |

**Table S9**. Species representation (NISP) by stratigraphic layer.

|  | **Very small**  **(rodents, birds, reptiles, and frogs)** | **Macromammals**  **(*Gazelle*, *Capra*, *Equus,* carnivore)** |
| --- | --- | --- |
| Layer 1 | 0 (0%) | 2 (100%) |
| Layer 2 | 1 (100%) | 0 (0%) |
| Layer 3 | 2 (40%) | 3 (60%) |
| Layer 4 | 1 (7%) | 14 (93%) |
| Layer 5 | 22 (88%) | 3 (12%) |
| Layer 6 | 1 (100%) | 0 (0%) |
| Layer 7 | 0 (0%) | 5 (100%) |
| Layer 8 | 16 (80%) | 4 (20%) |
| Layer 9 | 22 (96%) | 1 (4%) |
| Layer 10 | 20 (91%) | 2 (9%) |
| Layer 11 | 0 (0%) | 6 (100%) |
| Layer 13 | 0 (0%) | 1 (100%) |

**Table S10.** Fracture angle, outline, and edge data for long bone by animal size class

|  | **Microfauna** | **Macromammals** |
| --- | --- | --- |
| Fracture angle   - Oblique - Oblique/Right - Right | 29 (59%)  15 (31%)  5 (10%) | 11 (69%)  0 (0%)  5 (31%) |
| Fracture outline   - Curved/V-shaped - Intermediate - Transverse | 27 (53%)  14 (27%)  10 (20%) | 5 (31%)  7 (44%)  4 (25%) |
| Fracture edge   - Smooth - Jagged | 36 (77%)  11 (23%) | 3 (19%)  13 (81%) |
| Circumference type   - Type 1 - Type 2 - Type 3 | 22 (33%)  3 (4%)  42 (63%) | 10 (100%)  0 (0%)  0 (0%) |

**Table S11.** Results of corrosion analysis.

|  | **In situ molar digestion** | **Incisor digestion** |
| --- | --- | --- |
| Non-digested | 4 (67%) | 2 |
| Light | 1 (17%) | 1 |
| Moderate | 1 (17%) | 1 |
| Heavy | 0 (0%) | 0 |
| Extreme | 0 (0%) | 0 |


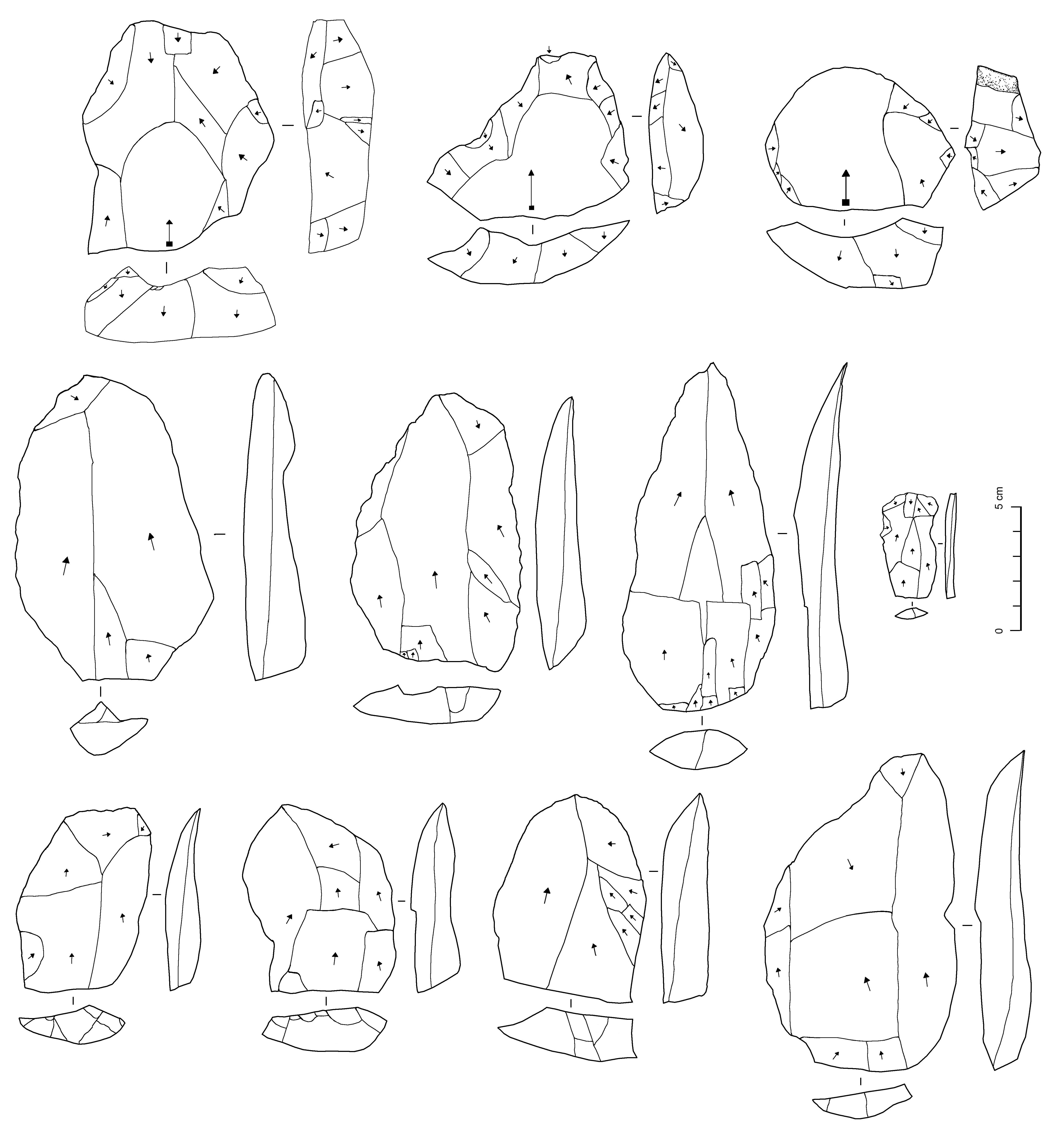


**Figure S4.** Obsidian lithics from Jebel Abyad. Top row: Levallois cores, bottom row: Levallois/Levallois-like flakes

**
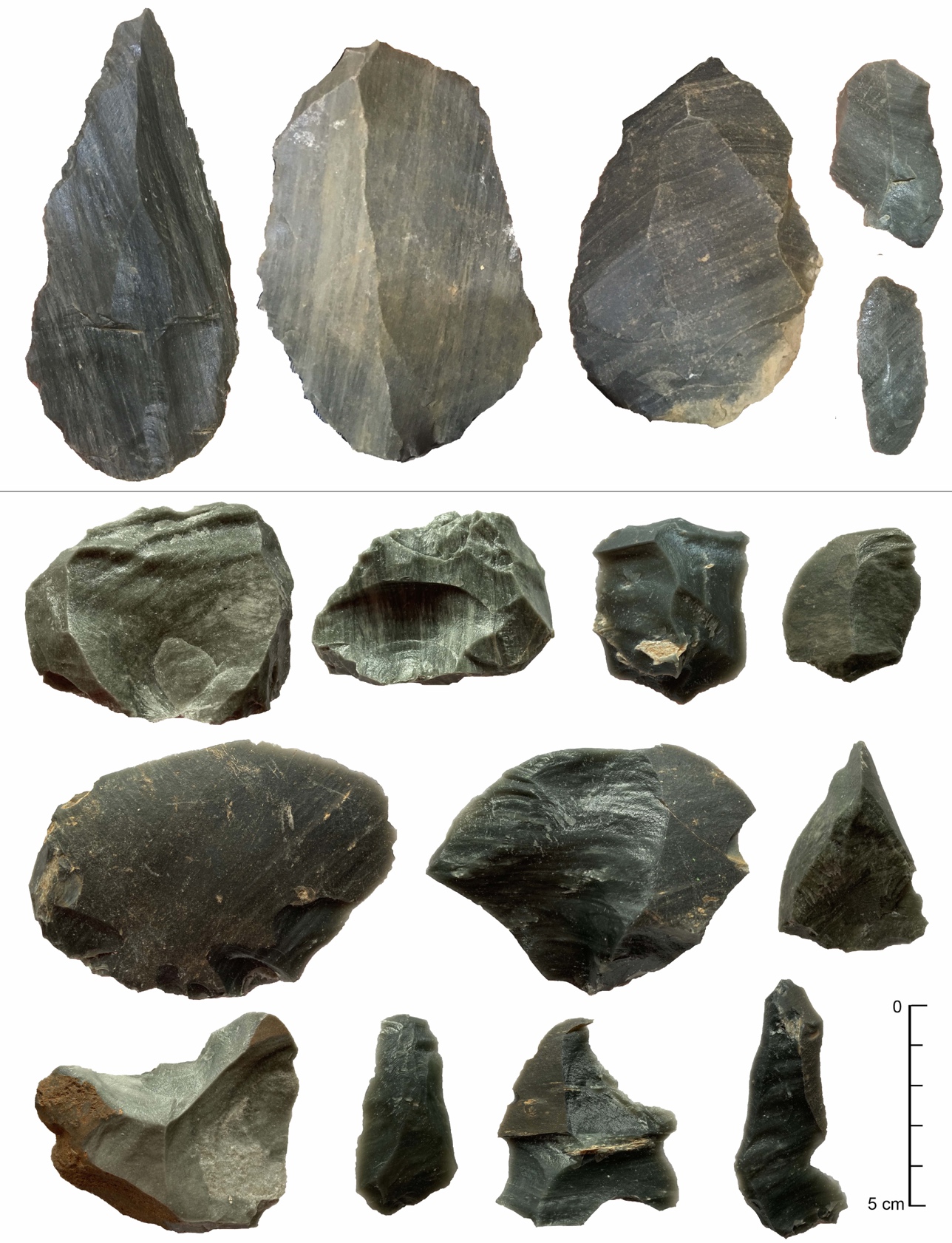
**

**Figure S5.** Lithics from localities 1–3 (top, above line), and experimentally produced stone tools (knapped by HG) using the same raw material at these sources.


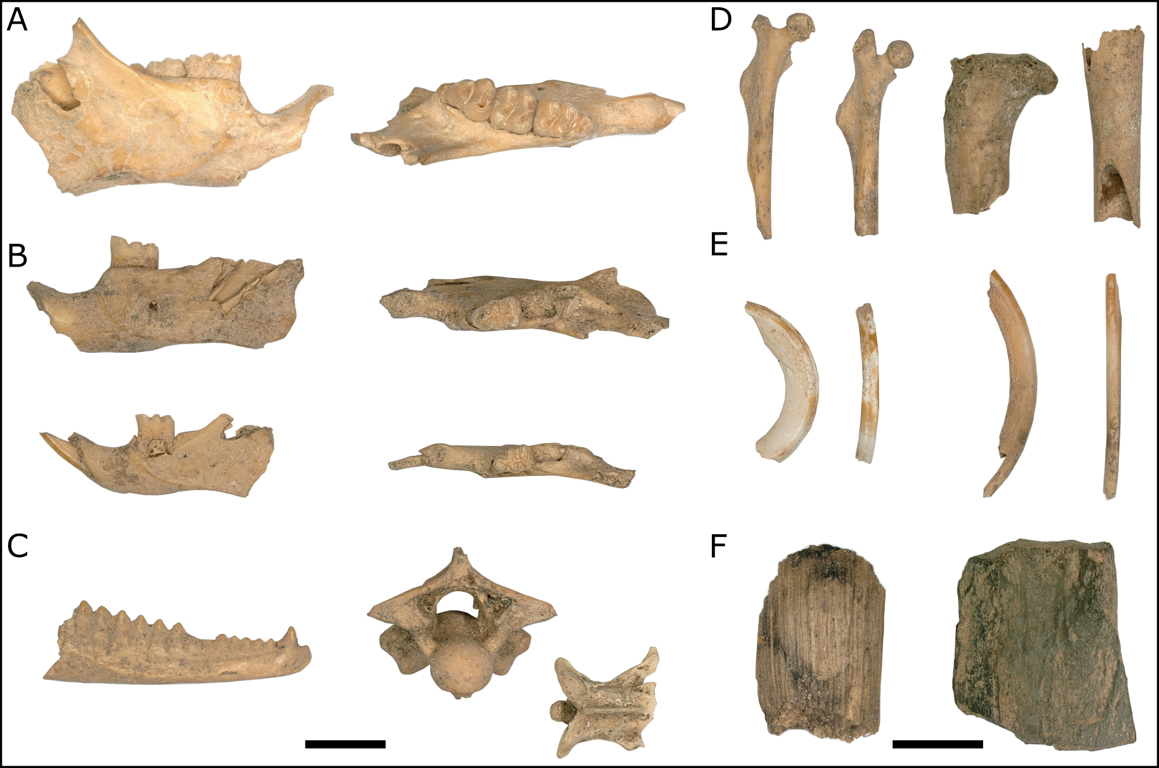


**Figure S6.** Examples of taxa and bone surface modifications. (A) Right mandible (UJS/5_05), cf. *Arvicanthus niloticus*; (B) two left mandibles (UJS/9_06, UJS/9_07), *Gerbillus*; (C) left mandible (UJS/10_10), Squamata; cervical vertebra (UJS/10_02, top left) Squamata; and vertebra (UJS/10_18), Serpentes; (D) light gastric corrosion on microfauna long bones; (E) moderate (left) and uncorroded (right) rodent incisors; (F) burned unidentified plants remains (left) and small-sized animal midshaft fragment (right). Scale bar on the left 4 mm, scale bar on the right 5 mm.


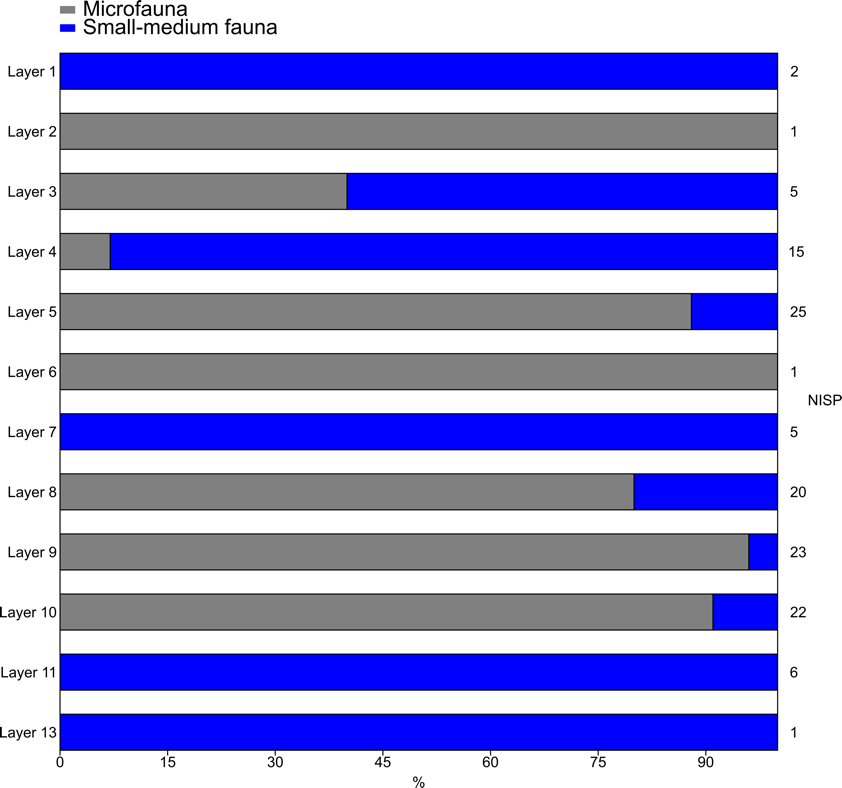


**Figure S7.** %NISP of microfauna and small- to medium-sized mammals by stratigraphic unit


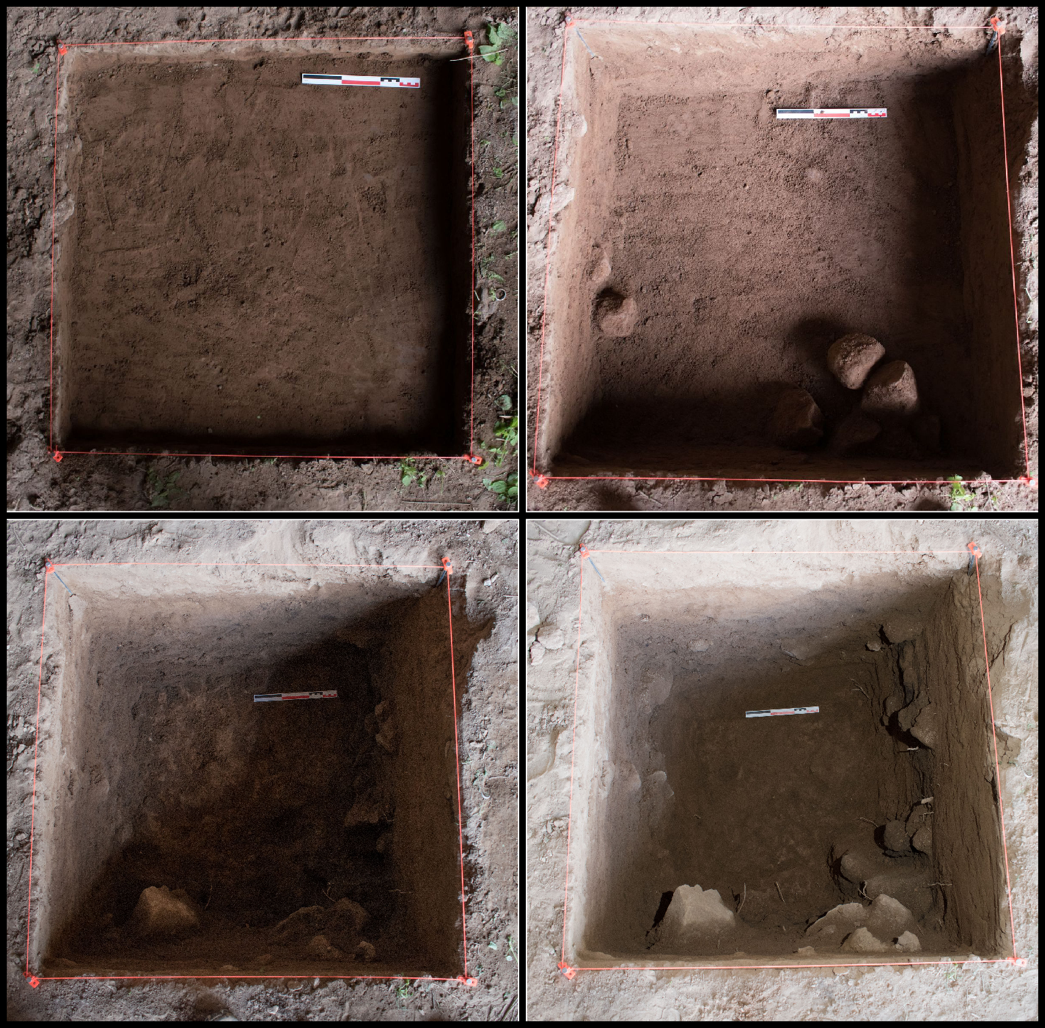


**Figure S8.** Overhead photos of the trench at various stages of excavation.


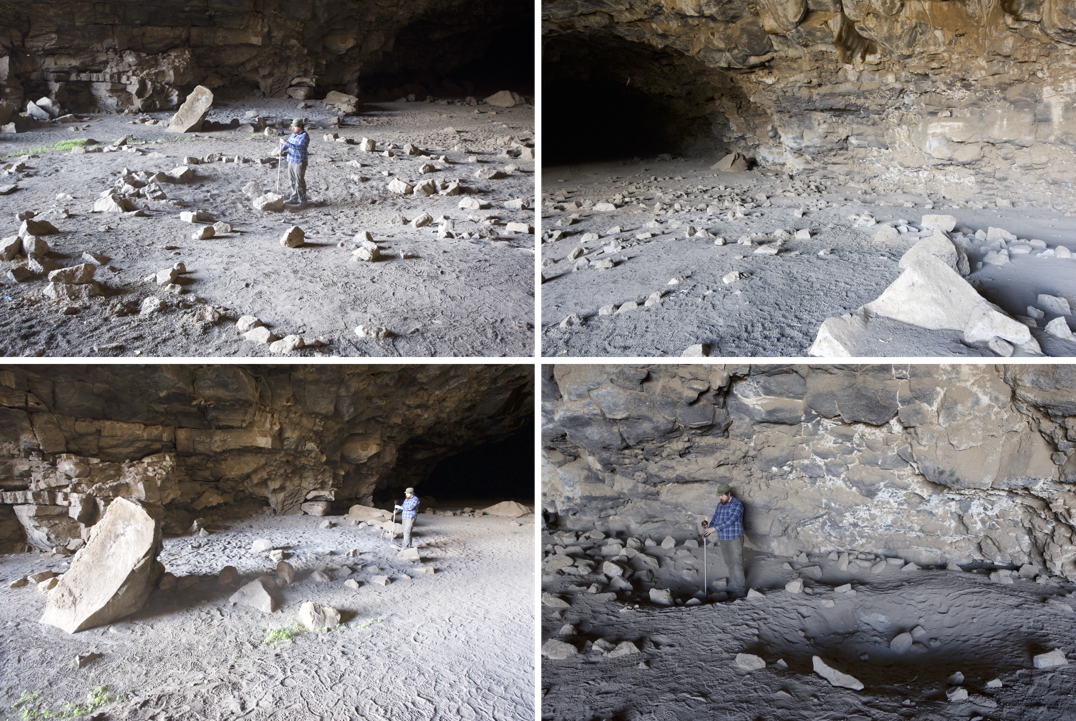


**Figure S9.** Examples of the Area C circular structures.

# Rock art

## Results

**Table S12.** Identifiable animals on each panel.

| Panel | sheep | goat | ibex | cattle | dog | bovid | quadruped | human figure |
| --- | --- | --- | --- | --- | --- | --- | --- | --- |
| 1 | 1 |  |  |  |  |  |  | 1 |
| 2 |  |  | 2 |  |  |  |  | 2 |
| 3 |  | 1 |  |  |  |  |  |  |
| 4 |  |  | 1 |  |  |  |  |  |
| 5 |  |  |  |  |  |  |  | ? |
| 6 |  | 2 | 2 | 1 |  | 1 | 1 | 1 |
| 7 | 1 |  | 2 |  |  | 2 | 1 | 2 |
| 8 | 5 | 3 | 2 |  | 2 |  | 2 | 6 |
| 9 | 9 |  |  |  | 1 |  |  | 1 |
| 10 | 1 |  |  |  |  |  | 1 |  |
| 11 | 1 |  |  |  |  |  | 1 |  |
| 12 | 1 |  |  |  |  |  | 1 |  |
| 13 |  | 1 |  |  |  | 1 |  |  |
| 13.5 | 1 |  |  |  |  |  | 1 |  |
| 14 | 3 |  | 6 |  |  | 1 |  |  |
| 15 |  |  |  | 1 |  |  | 1 |  |
| Total | **23** | **7** | **15** | **2** | **3** | **4** | **9** | **13** |

# References

Adamiec G and Aitken MJ (1998) Dose-rate conversion factors: update. *Ancient TL* 16: 37–46.

Aitken MJ (1985) *Thermoluminescence Dating*. London: Academic Press.

Armitage SJ and Bailey RM (2005) The measured dependence of laboratory beta dose rates on sample grain size. *Radiation Measurements* 39(2): 123–127: doi:10.1016/j.radmeas.2004.06.008.

Bailey RM and Arnold LJ (2006) Statistical modelling of single grain quartz De distributions and an assessment of procedures for estimating burial dose. *Quaternary Science Reviews* 25(19): 2475–2502: doi:10.1016/j.quascirev.2005.09.012.

Balescu S and Lamothe M (1994) Comparison of TL and IRSL age estimates of feldspar coarse grains from waterlain sediments. *Quaternary Science Reviews* 13(5): 437–444: doi:10.1016/0277-3791(94)90056-6.

Brennan BJ (2003) Beta doses to spherical grains. *Radiation Measurements* 37(4): 299–303: doi:10.1016/S1350-4487(03)00011-8.

Buylaert J-P, Jain M, Murray AS, Thomsen KJ, Thiel C and Sohbati R (2012) A robust feldspar luminescence dating method for Middle and Late Pleistocene sediments. *Boreas* 41(3): 435–451: doi:10.1111/j.1502-3885.2012.00248.x.

Buylaert J-P, Murray AS, Gebhardt AC, Sohbati R, Ohlendorf C, Thiel C, et al. (2013) Luminescence dating of the PASADO core 5022-1D from Laguna Potrok Aike (Argentina) using IRSL signals from feldspar. *Quaternary Science Reviews* 71: 70–80: doi:10.1016/j.quascirev.2013.03.018.

Duller GAT (2007) Assessing the error on equivalent dose estimates derived from single aliquot regenerative dose measurements. *Ancient TL* 25: 15–24.

Galbraith RF and Laslett GM (1993) Statistical models for mixed fission track ages. *Nuclear Tracks and Radiation Measurements* 21(4): 459–470: doi:10.1016/1359-0189(93)90185-C.

Galbraith RF, Roberts RG, Laslett GM, Yoshida H and Olley JM (1999) Optical dating of single and multiple grains of quartz from Jinmium Rock Shelter, northern Australia: part I, experimental design and statistical models. *Archaeometry* 41(2): 339–364: doi:10.1111/j.1475-4754.1999.tb00987.x.

Huntley DJ and Hancock RGV (2001) The Rb contents of the K-feldspar grains being measured in optical dating. *Ancient TL* 19: 43–46.

Li B, Jacobs Z, Roberts RG and Li S-H (2014) Review and assessment of the potential of post-IR IRSL dating methods to circumvent the problem of anomalous fading in feldspar luminescence. *Geochronometria* 41(3): 178–201: doi:10.2478/s13386-013-0160-3.

Lowick SE, Trauerstein M and Preusser F (2012) Testing the application of post IR-IRSL dating to fine grain waterlain sediments. *Quaternary Geochronology* 8: 33–40: doi:10.1016/j.quageo.2011.12.003.

Roberts HM (2012) Testing Post-IR IRSL protocols for minimising fading in feldspars, using Alaskan loess with independent chronological control. *Radiation Measurements* 47(9): 716–724: doi:10.1016/j.radmeas.2012.03.022.

Roberts RG, Galbraith RF, Yoshida H, Laslett GM and Olley JM (2000) Distinguishing dose populations in sediment mixtures: a test of single-grain optical dating procedures using mixtures of laboratory-dosed quartz. *Radiation Measurements* 32(5): 459–465: doi:10.1016/S1350-4487(00)00104-9.

Thiel C, Buylaert J-P, Murray A, Terhorst B, Hofer I, Tsukamoto S, et al. (2011) Luminescence dating of the Stratzing loess profile (Austria)–testing the potential of an elevated temperature post-IR IRSL protocol. *Quaternary International* 234(1): 23–31: doi:10.1016/j.quaint.2010.05.018.
